# ZEITLUPE Promotes ABA-Induced Stomatal Closure in Arabidopsis and *Populus*

**DOI:** 10.3389/fpls.2022.829121

**Published:** 2022-03-02

**Authors:** Manuela Jurca, Johan Sjölander, Cristian Ibáñez, Anastasia Matrosova, Mikael Johansson, Iwanka Kozarewa, Naoki Takata, Laszlo Bakó, Alex A. R. Webb, Maria Israelsson-Nordström, Maria E. Eriksson

**Affiliations:** ^1^Umeå Plant Science Centre, Department of Plant Physiology, Umeå University, Umeå, Sweden; ^2^Departamento de Biología Universidad de La Serena, La Serena, Chile; ^3^Umeå Plant Science Centre, Department of Forest Genetics and Plant Physiology, Swedish University of Agricultural Sciences, Umeå, Sweden; ^4^RNA Biology and Molecular Physiology, Faculty for Biology, Bielefeld University, Bielefeld, Germany; ^5^Forest Bio-Research Center, Forestry and Forest Products Research Institute, Hitachi, Japan; ^6^Department of Plant Sciences, University of Cambridge, Cambridge, United Kingdom

**Keywords:** abiotic stress, abscisic acid, circadian clock, stomatal closure, ZEITLUPE, OPEN STOMATA 1, PSEUDO-RESPONSE REGULATORs

## Abstract

Plants balance water availability with gas exchange and photosynthesis by controlling stomatal aperture. This control is regulated in part by the circadian clock, but it remains unclear how signalling pathways of daily rhythms are integrated into stress responses. The serine/threonine protein kinase OPEN STOMATA 1 (OST1) contributes to the regulation of stomatal closure *via* activation of S-type anion channels. OST1 also mediates gene regulation in response to ABA/drought stress. We show that ZEITLUPE (ZTL), a blue light photoreceptor and clock component, also regulates ABA-induced stomatal closure in *Arabidopsis thaliana*, establishing a link between clock and ABA-signalling pathways. ZTL sustains expression of *OST1* and ABA-signalling genes. Stomatal closure in response to ABA is reduced in *ztl* mutants, which maintain wider stomatal apertures and show higher rates of gas exchange and water loss than wild-type plants. Detached rosette leaf assays revealed a stronger water loss phenotype in *ztl-3*, *ost1-3* double mutants, indicating that ZTL and OST1 contributed synergistically to the control of stomatal aperture. Experimental studies of *Populus* sp., revealed that ZTL regulated the circadian clock and stomata, indicating ZTL function was similar in these trees and Arabidopsis. PSEUDO-RESPONSE REGULATOR 5 (PRR5), a known target of ZTL, affects ABA-induced responses, including stomatal regulation. Like ZTL, PRR5 interacted physically with OST1 and contributed to the integration of ABA responses with circadian clock signalling. This suggests a novel mechanism whereby the PRR proteins—which are expressed from dawn to dusk—interact with OST1 to mediate ABA-dependent plant responses to reduce water loss in time of stress.

## Introduction

As plants are sessile, their survival depends upon their ability to balance growth against stress mitigation. Plants must time their growth and modulate their water use on daily, seasonal and yearly timescales. Perennial plants, such as trees, may live for hundreds or even thousands of years (Burian et al., 2016) and must therefore inhibit growth under unfavourable conditions and manage a multitude of seasonal stresses over this lifespan. The timing of reproduction and growth is coordinated by the circadian clock, which uses light quality, photoperiod and temperature cues to entrain plants to local conditions (Millar, 2016). Arabidopsis (*Arabidopsis thaliana*) accessions coordinate germination and flowering with the seasonal patterns of their local environment, maximising survival, reproduction and seed yield (Green et al., 2002; Johansson et al., 2015; Rubin et al., 2017).

The plant hormone abscisic acid (ABA) controls many aspects of growth and development, including seed dormancy and germination, seedling growth and responses to abiotic and biotic stresses (Finkelstein, 2013). In *Populus*, ABA regulates seasonal growth (Tylewicz et al., 2018) and, in Arabidopsis, ABA controls cell growth by inhibiting the TARGET OF RAPAMYCIN (TOR) kinase (Wang et al., 2017).

Plants control water loss by opening or closing their stomata. Stomatal movements are therefore critical for balancing the conflicting needs of photosynthesis, gas exchange and water stress mitigation (Lawson and Vialet-Chabrand, 2018). In addition to its roles in controlling growth, ABA plays an important role in regulating stomata. ABA is produced during drought or light stress and evokes the local and systemic signals regulating stomatal aperture (Munemasa et al., 2015; Devireddy et al., 2018). In response to ABA, levels of osmotically active ions are reduced in guard cells, which leads to loss of turgor and stomatal closure (MacRobbie, 2000).

In the absence of stress, the PYRABACTIN RESISTANCE 1 (PYR1) / PYR1-LIKE (PYL)/REGULATORY COMPONENTS OF ABA RECEPTORS (RCAR) associates with type-2C protein phosphatases (PP2Cs), such as ABSCISIC ACID INSENSITIVE 1 (ABI1), ABI2, HYPERSENSITIVE TO ABA 1 (HAB1) or HAB2, in the cytosol to inhibit the two SUCROSE NONFERMENTING 1-related protein kinases SnRK2.2 (SRK2D), OPEN STOMATA 1 (OST1/SnRK2.6/SRK2E) and SnRK2.3 (SRK2I) (Baumann, 2010; Hubbard et al., 2010). Binding of ABA to PYR/PYL/RCAR (in the complex with PP2Cs) releases SnRK2s, thus enabling SnRK2 autophosphorylation and subsequent target protein phosphorylation (Vlad et al., 2010). Once activated, SnRK2s inhibit the inward rectifying ion channel K^+^ TRANSPORTER OF ARABIDOPSIS THALIANA 1 (KAT1) and activate the SLOW ANION CHANNEL-ASSOCIATED 1 (SLAC1) efflux of Cl^−^ and NO3^−^ anions (Hubbard et al., 2010). Calcium-dependent protein kinases activated by an ABA-induced elevation of cytosolic calcium are also required, phosphorylating and activating SLAC1 (Brandt et al., 2012, 2015; Munemasa et al., 2015). Therefore, the guard cell osmotic pressure is reduced, water is lost and stomata close. In the nucleus, OST1-dependent phosphorylation of b-ZIP transcription factors, such as ABSCISIC ACID INSENSITIVE3 (ABI3) and ABI5, mediates ABA-induced transcriptional change (Nakashima et al., 2009; Hubbard et al., 2010; Dai et al., 2013).

The circadian clock enables an organism to anticipate regular changes in its environment and modulate its development, growth, metabolism and even defence against predators (Sanchez and Kay, 2016). The plant circadian system is reset daily to local time *via* receptors detecting environmental cues of light and temperature (Millar, 2016), as well as by changes in metabolic sugar levels (Knight et al., 2008; Haydon et al., 2013; Shor et al., 2017). The plant oscillator consists of a large network of transcription factors of mainly repressive interlocking feedback transcription-translation circuits between the homologous proteins CIRCADIAN CLOCK ASSOCIATED 1 (CCA1), and LATE ELONGATED HYPOCOTYL (LHY), which show peak abundance in the morning, and TIMING OF CAB2 EXPRESSION 1 (TOC1)/PSEUDO-RESPONSE REGULATOR 1 (PRR1), which has an evening peak [see reviews (Farré and Liu, 2013; Millar, 2016; Sanchez and Kay, 2016; McClung, 2019)], and studies by Millar et al., (1995), Wang and Tobin (1998), Matsushika et al., (2000), Strayer et al., (2000), Alabadí et al., (2001), Locke et al., (2005), Gendron et al., (2012). These plant oscillator components are embedded in a wider network of interlocking feedback loops that ensure robust clock function with a period (cycle; *τ*) length close to 24 h (Fogelmark and Troein, 2014; Urquiza-García and Millar, 2021). The basic structure of the clock is conserved across plant species; thus, the clock of *Populus* spp. appears to function similarly to the ‘model’ clock developed from studies of Arabidopsis (Ramos et al., 2005; Zdepski et al., 2008; Takata et al., 2009; Hoffman et al., 2010; Ibáñez et al., 2010; Takata et al., 2010; Filichkin et al., 2011).

The period of the plant circadian oscillator is negatively related to the level of TOC1, whose phosphorylation and nuclear import are modulated by PRR5 (Millar et al., 1995; Strayer et al., 2000; Eriksson et al., 2003; Más et al., 2003; Fujiwara et al., 2008; Wang et al., 2010). ZEITLUPE (ZTL), a central F-box clock protein and blue light receptor, acts in an E3-ligase complex that controls proteasomal degradation of both TOC1 and a structurally similar protein, PRR5, that is expressed slightly earlier in the day (Han et al., 2004; Somers et al., 2004; Kevei et al., 2006; Kiba et al., 2007; Fujiwara et al., 2008). ZTL levels show a rhythmic pattern over 24 h, with troughs and peaks occurring near dawn (i.e. lights-on in a controlled environment) and dusk (lights-off), respectively (Kim et al., 2007; Lee et al., 2018), as a result of its interaction with GIGANTEA (GI). GI stabilises ZTL *in vivo* by a direct protein-protein interaction *via* the amino-terminal flavin-binding LIGHT, OXYGEN OR VOLTAGE (LOV) domain. This interaction is stabilised by blue light. Mutations within the LOV domain, such as *ztl-21* (Kevei et al., 2006), significantly reduce the interaction between ZTL and GI, decreasing ZTL levels (Kim et al., 2007). GI also recruits deubiquitylases to modulate ZTL-complex function (Lee et al., 2019).

Daily rhythms of stomatal aperture are subject to direct regulation by light *via* blue and red light photoreceptors that control the activities of H^+^-ATPase and other ion channels (Hubbard et al., 2010). Multiple signals, such as ABA, CO_2_ and extracellular calcium, converge to control stomatal guard cells (Webb and Hetherington, 1997; Israelsson et al., 2006). ABA-signal transduction initiates stomatal closing and inhibits stomatal opening. The Arabidopsis circadian mutant *toc1-1* has a short period rhythm of stomatal opening under constant conditions, indicating the involvement of the circadian clock in regulating stomatal aperture (Somers et al., 1998); conversely, a long period *ztl-1* mutation delays the daily rhythms of carbon assimilation and stomatal conductance (Dodd et al., 2004), and also affects water use efficiency (Simon et al., 2020).

Mathematical modelling suggests the circadian period changes upon application of ABA (Pokhilko et al., 2013). Experimentally, this prediction is supported by the finding that MYB96 feeds back into the oscillator through the transcriptional activation of TOC1 (Lee et al., 2016).

Stress responses involving the induction of ABA interact in several ways with circadian signalling pathways. Interestingly, the clock protein PRR5 contributes to ABA regulation and signalling, as well as to several ABA-regulated responses, and was recently shown to promote germination synergistically with ABI5 in the presence of ABA (Yang et al., 2021). Overexpression of PRR5 enhances the effect of ABA signalling, inhibiting seed germination in the presence of ABA, while underexpression reduces it.

We addressed the integration of the circadian clock with ABA-dependent stress responses by testing ABA-induced stomatal closure. We reasoned that stomatal closure in the evening was critical to stress and growth regulation, and thus assayed Arabidopsis plants carrying mutations in evening-expressed circadian clock proteins. Consistent with earlier findings (Dodd et al., 2004; Simon et al., 2020), mutations at the *ZTL* locus significantly affected stomatal closure. We also established that ZTL-mediated regulation of stomata was conserved across species by analysing stomata in **P*opulus *t*remula* L. *× P. *t*remuloides* Michx. (*Populus; Ptt*) lines with reduced expression of *PttZTL* orthologues. The role of ZTL in ABA-induced signalling was investigated; it showed that ZTL function is necessary to sustain expression of *AREB/ABF/ABI5* gene expression in response to ABA.

Stomatal movements in both species indicated that the circadian clock acted *via* ZTL to modulate water use efficiency (Simon et al., 2020) and ABA signalling (Adams et al., 2018). We therefore undertook further genetic, physiological and biochemical analyses to determine if an interaction between ZTL and OST1 controlled stomatal closure. ZTL and OST1 interacted physically in plant cells. ABA-induced gene expression in the Arabidopsis mutant *ztl-3* resembled the effect of the strong *ost1-3* allele (Mustilli et al., 2002).

As ZTL post-translationally regulates PRR5, changes to diel regulation of stomata as well as their stress regulation and may be affected by accumulation of PRR5. Hence, we examined stomatal aperture and water loss in *prr5* mutants. Stomatal aperture was affected by loss of PRR5, and PRR5 could interact with OST1 in plant cells. *ztl-3 and ost1-3* double mutants showed an increased sensitivity to drought compared to both single mutants, suggesting these proteins acted in a synergistic manner and may be affected by PRR5 accumulation. Studies of their genetic interactions showed that PRR5 was involved in stomatal closure and suggested the circadian and ABA-signalling pathways converged at ZTL and the control of PRR5, with both proteins interacting with OST1 to control stomatal movements. The circadian signalling and ABA-stress response pathways are thus closely integrated, each modulating the other to enable plants to balance the trade-off between managing growth and mitigating environmental stress in a timely manner.

## Materials and Methods

### Arabidopsis Germplasm

Seeds of Arabidopsis [*Arabidopsis thaliana* (L.) Heynh.] containing the T-DNA insertion allele *ztl-3* (SALK_035701) in the Columbia-0 (Col-0) background (Jarillo et al., 2001; Kim et al., 2003) were obtained from the Salk collection^1^ at the Arabidopsis Biological Resource Centre (ABRC), Ohio State University, Ohio, United States *via* the Nottingham Arabidopsis Stock Centre (NASC), Nottingham, United Kingdom. Seed of the *ost1-3*/*snrk2.6*/*srk2e* mutant (SALK_008068; Yoshida et al., 2002) was provided by Dr. Kazuko Yamaguchi-Shinozaki (University of Tokyo, Japan) to the Israelsson-Nordström laboratory. The Webb laboratory received seed of the *ztl-4, fkf1-2 and lkp2-1* triple mutant (Baudry et al., 2010) from Dr. Steve Kay (Keck School of Medicine, University of Southern California, United States) and of the *prr5-11* mutant (Nakamichi et al., 2005) from Dr. Takeshi Mizuno (Nagoya University, Japan). The *ztl-1* mutant in the C24 background, the *ztl-21* mutant in the Wassilewskija-2 (Ws-2) background and *prr5-1* (SALK_006280) have all been described previously (Eriksson et al., 2003; Kevei et al., 2006; Kiba et al., 2007).

The *ztl-3 and ost1-3* double mutant in the Col-0 background were generated using *ztl-3* as the pollen recipient and *ost1-3* as the pollen donor. The *ztl-3, prr5-1 and ost1-3* triple mutant, and combinations thereof, were generated using *ztl-3; prr5-1* (Norén et al., 2016) as the pollen recipient and *ost1-3* as the pollen donor.

To complement the loss-of-function mutant *ztl-3*, we generated transgenic *ztl-3* plants expressing *ZTL* under the control of the 35S CaMV constitutive promoter. *Agrobacterium tumefaciens* GV3101 (pMP90RK-pSoup) was transformed with the *p35S::HA::ZTL* construct in pGreenII 0229. The construct was introduced into *ztl-3* mutant Arabidopsis plants by floral dipping (Bechtold et al., 1993). The pGreenII 0229 construct contains the bialaphos resistance gene (*BAR*) that confers resistance to glufosinate-ammonium (Hellens et al., 2000). Seeds from dipped plants were sterilised and plated on full-strength Murashige and Skoog (MS) medium supplemented with vitamins (Duchefa, BH Haarlem, Netherlands), 3% w/v sucrose (SIGMA-Aldrich, Saint Louis, MO, United States) and 0.8% agar (E1674, Duchefa), pH 5.7, plus 10 mg/l glufosinate-ammonium (SIGMA-Aldrich). Resistant plants were allowed to self-fertilise and next-generation seeds were again screened on glufosinate-ammonium. Expression of the *p35S::HA::ZTL* construct was confirmed by Western blotting (not shown), prior to phenotyping.

### Radicle Emergence Assay

Radicle emergence assays were conducted using seeds sown on plates containing half-strength MS medium with 0.8% agar, pH 5.7. Seeds of the different genotypes were grown and harvested at the same time under the same conditions and stored for least 3 months after harvest as seed age and storage affect germination responses. Seeds were surface-sterilised by consecutive washes with 15% hypochlorite, 70% ethanol with 0.1% tween and 95% ethanol before plating. Plated seeds (60 seeds per genotype per replicate; Supplementary Figure S1) were stratified at 4°C for 2 days and transferred to a growth chamber (LD 16:8 at 22°C; light intensity during day: 150 μmol m^−2^ s^−1^) to initiate germination. Germination was determined by the appearance of the testa, endosperm rupture and radicle protrusion (Wu et al., 2012). Radicle emergence was scored every 12 h, starting at 24 h, and calculated as a percentage of the plated seeds.

### *Populus* Material and Growth Assays

*In vitro*-cultivated, rooted cuttings of *PttZTL1,2* RNA interference (RNAi) lines and wild-type (WT) **P*opulus *t*remula* L. × *P. *t*remuloides* Michx. cv. T89 (Nilsson et al., 1992; *Populus; Ptt*) plants were potted in a 3:1 mix of fertilised peat and perlite, and established under long day cycles consisting of light:dark (LD) 18:6 at constant temperature (18°C) and 80% relative humidity for 4 weeks; light intensity during the day was 200 μmol m-^2^ s^−1^ (Osram Powerstar HQI-T 400 W/D lamps; Osram, München, Tyskland). After this point, temperature, humidity and irradiance during the day were maintained but the photoperiod was shortened to LD 15:9 at 18°C, keeping the time of dawn unchanged.

A sub-set of lines (1, 3, 4, 5 and 7) with variable levels of *ZTL1,2* downregulation were selected and samples for analysis taken twice a week subsequent to the shift from LD 18:6 to LD 15:9. Growth cessation, which refers to the elongation of shoots ceasing, was scored according to a predefined scale (score 2), as previously described (Ibáñez et al., 2010).

### Gene Constructs

#### *Populus* ZTL RNA Interference

The *ZTL* RNAi fragment from *Populus* was used to construct RNAi lines; this fragment targets two homologous genes, *PttZTL1* and *PttZTL2* [Potrx050857g15511 and Potrx063764g24087 (Sjödin et al., 2009)].^2^ Template cDNA from wild-type (WT) *Populus* was amplified by PCR using Platinum Pfx DNA polymerase (Invitrogen, Carlsbad, CA, United States) and gene-specific primers (Supplementary Material).

The amplified fragment was cloned into the Gateway entry vector pDONOR201, followed by recombination into the binary vector pHELLSGATE8 (Helliwell et al., 2002) using Gateway BP Clonase enzyme mix (Invitrogen, Carlsbad, CA, United States). WT trees were transformed with the resulting binary vector that contained the RNAi construct, using *Agrobacterium tumefaciens* C58 strain GV3101 (pMP90RK; Nilsson et al., 1992, 1996). Transgenic plants were selected using kanamycin and regenerated, as described previously (Eriksson et al., 2000). This produced 10 independent, stable and first-generation *PttZTL1,2* RNAi transgenic lines.

RNA was extracted from a pool of leaves collected from the 10th internode of different *PttZTL1,2* RNAi lines; leaf samples were collected at five time points from trees of each line, with one leaf sampled every 4 h, starting at ZT 0 (dawn) under a 24 h cycle (Supplementary Figure S2A) or pools from the 8, 9 and 10th internode collected at ZT 12 (Supplementary Figure S2B). An RT-qPCR analysis with gene-specific primers (Supplementary Material) determined the extent of downregulation of *PttZTL1* and *PttZTL2* in each independent line.

#### Arabidopsis ZTL Over Expression Construct

To produce constructs overexpressing ZTL, the *ZTL* coding sequence under the control of the 35S CaMV promoter and fused in frame with the 3 × HA-epitope tag (*p35S::HA::ZTL*) was obtained from pRT104-HA-ZTL (Johansson et al., 2011) by SbfI restriction digest. The *p35S::HA::ZTL* fragment was then subcloned into the PstI site of the promoter-less pGreenII 0229 plasmid (Hellens et al., 2000). Positive colonies were selected by colony PCR using primers that amplified the ZTL ORF. The cloned construct was sequenced and used to transform competent *A. tumefaciens* GV3101 (pMP90RK-pSoup). *A. tumefaciens* clones containing the *p35S::HA::ZTL* construct were selected in medium containing 25 μg/ml kanamycin and confirmed by colony PCR; a single confirmed clone was used to transform Arabidopsis plants *via* the floral dip method (Bechtold et al., 1993). Independent lines of T_3_ transgenic seeds were used in experiments.

#### Delayed Fluorescence of *Populus* and Period Analysis

Young leaves from internodes four to six were dissected from sterile cuttings grown in jars on half-strength Murashige and Skoog (MS) medium, supplemented with vitamins (Duchefa) and 0.8% agar (E1674, Duchefa), pH 5.7, under 18 h light:6 h dark (LD 18:6) cycles. The light intensity during the day was 100–120 μmolm^−2^ s^−1^. The light:dark cycle was reinforced by warm:cold (W:C) temperature cycles (20°C during light:18°C during dark).

Excised leaves, together with a clean-cut petiole to sustain growth, were placed on square plates (12 × 12 cm) containing MS medium. Plates were transferred at Zeitgeber time (ZT) 0 (i.e. ‘dawn’ or lights-on) to constant light (LL), consisting of equal parts blue (470 nm) and red light (660 nm) from 20 μmolm^−2^ s^−1^ light-emitting diodes (MD Electronics, Warwick, United Kingdom) at a constant temperature of 22°C. Rhythms of delayed fluorescence were recorded following lights-off using a cooled ORCA-IIERG 1024 camera (Hamamatsu Photonics, Hamamatsu City, Japan) with medium gain, a 900 ms delay and 1 min exposure. Imaging data were analysed using BRASS Fourier analysis software (Plautz et al., 1997; Locke et al., 2005), as described previously (Ibáñez et al., 2010). Only data collected 24–120 h after the transfer to LL were included in the analysis. Plants were considered rhythmic when the relative amplitude error was ≤0.6.

#### Water Loss Assay

Water loss assays were performed in detached leaves of similar developmental stage and size from 3-week-old Arabidopsis or 12- to 13-week-old *Populus* plants. Arabidopsis plants were grown on soil under controlled conditions (LD 16:8 cycles; light intensity during the day: 100–120 μmol m^−2^ s^−1^) at 22°C. For Arabidopsis assays, a single leaf per plant (six to eight plants per genotype) was detached and the loss in fresh weight monitored over time. For *Populu*s, plants from WT and RNAi lines were grown under controlled conditions (LD 18:6 cycles; light intensity during the day: 250 μmol m^−2^ s^−1^) at 18°C. Three expanded young leaves from internodes eight to 10 (counted from the first leaf at least 1 cm long) were detached from each plant (six plants per genotype) and the loss in fresh weight monitored over time. Water loss was expressed as a percentage of the initial fresh weight.

#### Stomatal Conductance

Arabidopsis and *Populus* plants were grown as described for the water loss assay. Stomatal conductance (gs = mmol H_2_O m^−2^ s^−1^) was measured in intact 3-week-old wild-type, *ztl-3* and *ost1-3* Arabidopsis plants using a steady-state Leaf Porometer (Decagon Devices, Pullman, United States). Measurements were made on the abaxial leaf surface from four leaves per plant, and from eight to 10 plants per genotype. Conductance was measured in *Populus* in three expanded young leaves from internodes eight to 10, as defined above, and from seven to eight plants per genotype.

#### Stomatal Aperture and Density Measurements

The stomatal ratio is a better measure than aperture as it accounts for differences in stomatal size that may occur in expanding leaves with different growth rates. To calculate the stomatal ratio, measurements of stomata opening were made, as previously described (Conn et al., 2011). Plants were cultivated *in vitro* in Petri dishes (Arabidopsis) or in jars (*Populus*) for 3 to 4 weeks on half-strength MS with 0.8% agar, pH 5.7, under LD 16:8 (*Populus*) or LD 12:12 (Arabidopsis) at 20°C, light intensity during day ~200 μmol m^−2^ s^−1^. Leaves were collected and transferred to 20 ml 10 mM MES pH 6.2 (adjusted with KOH) and blended in pulses (3 × 30 s) with 10 s intervals between pulses. The suspension was filtered through a mesh (pore size: 100 μm) and epidermal fragments were collected in 10 ml 10 mM MES. Samples were incubated in the dark for 1 h at 20°C before the epidermal fragments were filtered and collected in 20 ml buffer (5 mM KCl, 0.1 mM CaCl_2_ in 10 mM MES pH 6.2). The samples were divided into two equal parts; 10 μl 10 mM ABA dissolved in 70% ethanol was added to one part and 10 μl 70% ethanol to the other as a control (both aliquots were diluted in 10 ml 10 mM MES). Both treatment and control samples were incubated for 3 h in a water bath at 20°C under cold fluorescence light and CO_2_-free aeration (Conn et al., 2011). Measurements were made on stomata from plants treated either at ZT 8–9 or at a time adjusted to circadian time (CT) 9 for each genotype to accommodate the longer circadian period (~28–29 h) of *ztl-3* (Somers et al., 2004) and shorter (~22–23 h) period of *prr5-11* (Yamamoto et al., 2003); the circadian phenotype of *prr5-11* is comparable to *prr5-1* (Eriksson et al., 2003; Michael et al., 2003). In each case, the experimenter was blinded to genotype and treatment until the analysis was completed. Plant tissues were incubated in the presence/absence of ABA for 3 h in light, under CO_2_-free aeration (Conn et al., 2011), and stomatal closure was scored at ZT 8–9, i.e. 8–9 h after dawn.

Stomatal density was measured in leaves from 3- to 4-week-old soil-grown Arabidopsis plants cultivated under long photoperiods (LD 16:8) and concomitant temperature cycles (WC 20°C:18°C). Plants were grown and treated in the same manner as the plants subjected to stomatal conductance measurements, described above. Measurements were made from one leaf per plant and from eight to 10 plants per genotype. Stomatal density in *Populus* wild type and RNAi lines was measured in fully expanded young leaves from internodes eight to 10 from soil-grown plants cultivated under long photoperiods (LD 18:6) at 18°C. For density measurements in both species, the abaxial surface of leaves from three plants per genotype was peeled off and visualised using an Axiocam digital camera attached to an Axioplan light microscope (Carl Zeiss Microscopy GmbH, Oberkochen, Germany). The number of stomata per mm^2^ of leaf area was counted using the software provided.

#### Protein Expression Constructs

Expression vectors to express epitope-tagged proteins in protoplasts were obtained by cloning full length coding sequences into pRT104 plasmids carrying 3 × HA or 3 × Myc epitopes under the constitutive CaMV35S promoter (Fülöp et al., 2005). Epitope-tagged ZTL and TOC1 were obtained as described previously (Johansson et al., 2011).

To obtain epitope-tagged OST1 and PRR5, the full length cDNAs clones were obtained from ABRC,^3^ inserted into either pENTR D-TOPO (*OST1*) or pUNI51 (*PRR5*), and used as templates for PCR. The OST1 PCR amplified coding sequence was cloned into pRT104 at the EcoRI/SalI sites and PRR5 at the BamHI/KpnI sites.

All constructs were confirmed by sequencing before use. The primer sequences are listed in Supplementary Material.

#### Transient Protoplast Expression Assays

An Arabidopsis Col-0 cell suspension culture was grown under LD 16:8 with concomitant WC 22°C:18°C cycles; light intensity during day: 150 μmol m^−2^ s^−1^. Protoplasts were co-transfected with HA or Myc-tagged proteins in the combinations ZTL + OST1, ZTL + TOC1 and OST1 + PRR5 prior to co-immunoprecipitation assays (Meskiene et al., 2003). Protoplasts were harvested for protein extraction 18 h post-transfection and suspended in immunoprecipitation buffer [25 mM Tris–HCl, pH 7.8, 10 mM MgCl_2_, 75 mm NaCl, 5 mM EGTA, 60 mM β-glycerophosphate, 1 mM dithiothreitol, 10% glycerol, 0.2% Igepal CA-630 and 1× Protein Inhibitor Cocktail (SIGMA-Aldrich)]. The samples were frozen in liquid nitrogen until use.

For immuno-analysis, samples were thawed on ice and centrifuged. Supernatants were mixed with 1.5 μl 5 M NaCl, 1.5 μl anti-Myc antibody (9E10; Covance, Princeton, NJ, United States), 1 μl 20 mg/ml BSA and immunoprecipitation buffer to a final volume of 100 μl. The mixtures were incubated for 2 h at 4°C on a rotating wheel. Immune complexes were captured by adding 10 μl Protein G-Sepharose beads (Wu et al., 2009) to the mixtures and incubating for a further 2 h at 4°C with rotation. The beads were washed three times with ice-cold immunoprecipitation wash buffer (25 mM sodium phosphate, 150 mM NaCl, 5% glycerol and 0.2% Igepal CA-630). Immune complexes were eluted from beads using 25 μl 1× SDS buffer. Proteins were separated by electrophoresis on 8% SDS polyacrylamide gels and blotted into Immobilon-P PVDF membranes (Millipore Corporation, Billerica, MA, United States). The presence of HA-tagged proteins in the immune complexes was determined by probing blots with anti-HA-POD antibody (3F10; Roche Diagnostics, Basel, Switzerland). Subsequently, blots were stripped for 15 min at 70°C in buffer (100 mM β-mercaptoethanol, 2% SDS and 62.5 mM TRIS pH 6.8) and incubated with anti-c-Myc chicken antibody (A21281; Thermo Fisher Scientific) to confirm the presence of Myc-tagged proteins in the complex. Co-IP results, where quantified, were measured using ImageJ software (Schneider et al., 2012). Measurements of immunoprecipitated HA-tagged protein bands were made on acquired images in ImageJ after background subtraction. The values indicated are the ratio of the HA-tagged pull-down protein density to input samples, with loaded input ˗40% of the total reaction. The results are average of three independent experiments.

Mesophyll protoplasts were isolated according to the tape-Arabidopsis sandwich procedure (Wu et al., 2009). Plants were grown as described for the water loss assay and 8–10 leaves were collected from 3- to 4-week-old plants. Protoplasts were transfected using a modified TEAMP to investigate whether binding method (Yoo et al., 2007). For protein stability assays, transfected protoplasts were incubated for 3 h with 100 μM cycloheximide (CHX), an inhibitor of protein synthesis, before sample collection at the indicated time points. Monoclonal anti-PSTAIR CDKA antibody was hybridised at 1:5000 dilution (SIGMA-Aldrich). The protein stability assays in mesophyll protoplasts were performed using 50 μM MG132 (SIGMA-Aldrich), a proteasome inhibitor. Protoplasts were incubated with MG132 or DMSO (control) for 4 h. An antibody detecting the RUBISCO small subunit antibody (AS07 259, Agrisera AB, Vännäs, Sweden) was used as a loading control.

#### Quantitative Reverse Transcription PCR

To characterise *PttZTL* RNAi lines, plants were grown in a greenhouse under controlled conditions (LD 18:6 at 18°C; light intensity during day: 250 μmol m^−2^ s^−1^). Leaf samples were collected 8 h after dawn (ZT 8) and frozen in liquid nitrogen. RNA was extracted from one fully developed leaf per sample, using the CTAB method (Le Provost et al., 2007), and treated with DNase (TURBO DNA-free kit; Ambion, Austin, United States). cDNA was synthesised from 1 μg RNA using the iScript cDNA Synthesis Kit (Bio-Rad Laboratories). Quantitative reverse transcription PCR (RT-qPCR) was performed using a CFX96 Real-Time detection system (Bio-Rad Laboratories) and gene-specific primers (Supplementary Material).

The increase in SYBR Green fluorescence (Bio-Rad Laboratories) was used to visualise the accumulation of PCR products in real time. All RT-qPCR reactions were performed using three or four biological repetitions with duplicate technical samples. *PttZTL* expression levels were normalised against expression of the reference genes *ELONGATION FACTOR 1 ALPHA* (*EF1a*) or 18S rRNA using the 2^−ΔΔCT^ method incorporating the primer efficiencies obtained by experiment, which ranged from 98 to 100% (Livak and Schmittgen, 2001; Pfaffl, 2001). Expression data are presented relative to expression in wild-type *Populus*. Data were log2 transformed to obtain a normal distribution and analysed using Student’s *t*-test. Primer sequences are listed (Supplementary Material); primers for *PttZTL* amplified both *ZTL* homologues. For gene expression analyses in Arabidopsis, seeds were surface-sterilised, plated *in vitro* and grown as described above for 3–4 weeks. Seedlings were sprayed with 10 μM ABA (10 μl ABA stock dissolved in 70% ethanol) and diluted in 10 ml water or control (equal amount of 70% etanol dissolved in water) 3 h prior to harvesting at ZT 8. Gene expression levels were determined using RT-qPCR, as described above, with gene-specific primers (Supplementary Material). Expression of each gene was normalised against expression of *EF1a* using the 2^−ΔΔCT^ method (Livak and Schmittgen, 2001; Pfaffl, 2001). The efficiency of each primer pair was included in the analysis (efficiency ranged from 95.3 to 100%). Gene expression levels are shown relative to expression in wild-type plants without ABA treatment, which was set as 1. Log2-transformed values were analysed by two-way ANOVA followed by Sidak’s multiple comparison test.

#### Statistical Analyses

The statistical significance of results was tested using one- or two-way ANOVA followed by *post-hoc* comparisons (Tukey’s test or Sidak’s test, each corrected for multiple comparisons) or unpaired Student’s *t*-tests, as indicated, using GraphPad Prism version 6.0 for Windows (GraphPad Software, La Jolla, CA, United States). In each case, significance was taken at alpha <0.05.

## Results

### ABA-Induced Stomatal Closure Is Impaired in *ztl* Mutants

**Figure 1:**
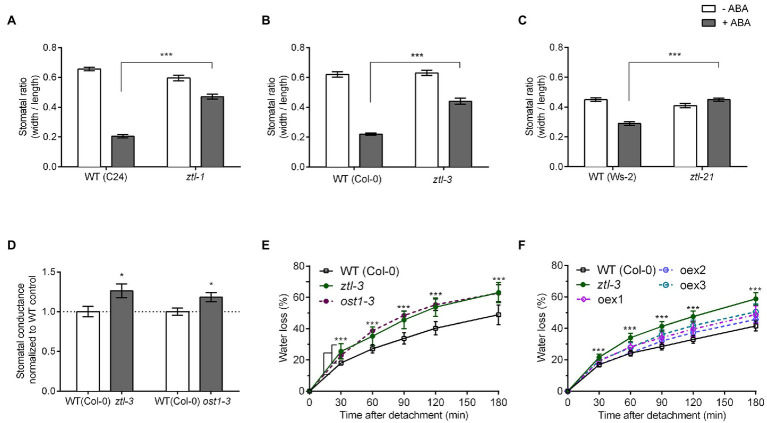
Stomatal opening and water loss phenotypes of detached Arabidopsis leaves. **(A–C)** Stomatal aperture in epidermal strips from leaves of 4-week-old seedlings treated with or without 10 μM ABA measured at ZT 8–9 under CO_2_-free aeration. **(A)** *ztl-1* and WT (C24) plants; **(B)** *ztl-3* and WT (Col-0); and **(C)** *ztl-21* and WT (Ws-2). **(D)** Stomatal conductance (gs) in leaves of 4-week-old Arabidopsis plants. Conductance was normalised against the WT means. **(E,F)** Rates of water loss in detached rosette leaves from 3-week-old Arabidopsis plants. Values are means ± SE of three biological replicates, each containing one leaf from six to eight plants of each genotype. **(E)** Rates of water loss in WT (Col-0); *ztl-3* and *ost1-3*. Asterisks in **(E)** refer to both the comparison between WT and *ztl-3* and between WT and *ost1-3* (Student’s *t*-test); both mutants showed the same level of statistical difference from WT. **(F)** Rates of water loss in WT (Col-0); *ztl-3* and *ztl-3:p35S::HA::ZTL* (oex1-3: independent lines of *ztl-3* overexpressing ZTL). Asterisks in **(F)** represent the comparison between WT and *ztl-3* plants (Student’s *t*-test); water loss from all other mutants did not differ statistically from that of WT plants. WT and *ztl-3* plants (Student’s *t*-test); water loss from all other mutants did not differ statistically from that of WT plants. **(A–F)** ^*^p < 0.05 and ^***^p < 0.001 (Student’s *t*-test).

In Arabidopsis, sensitivity to ABA, which induces stomatal closure, increases in the late afternoon (Correia and Pereira, 1995). This timing correlates with high levels of ZTL (Kim et al., 2007). We therefore investigated the sensitivity of *ztl* mutants to ABA. As defects in the different protein domains affect different aspects of ZTL function (Kevei et al., 2006; Kim et al., 2007), we measured sensitivity to ABA in *ztl-3* (complete loss-of-function mutant), *ztl-1* (Kelch domain mutant) and *ztl-21* (LOV domain mutant). Although ABA evoked stomatal closure in *ztl* mutants, the response was significantly greater in wild-type (WT) plants (Figures 1A–C).

### Mutations in *ZTL* and *OST1* Have Similar Effects

We measured stomatal conductance in *ztl-3* and *ost1-3* leaves using a handheld SC-1 leaf porometer (Decagon). Stomatal conductance levels were higher in *ztl-3* and *ost1-3* than in WT controls (Figure 1D). *ost1-3* mutants have the same stomatal density as WT plants (Engineer et al., 2014; Jalakas et al., 2018). Stomatal density was similar in WT (208 stomata/mm^2^) and *ztl-3* plants (210 stomata/mm^2^; Student’s *t*-test *p* = 0.727, *n* = 3 biological replicates, each containing 71–93 measurements/genotype). Thus, the increased water loss of *ztl-3* and *ost1-3* mutants was not caused by an increase in the number of stomata. The higher stomatal conductance in *ztl-3* (Figure 1D) observed is consistent with their reduced response to ABA (Figures 1A–C). Overall, the ABA sensitivity and stomatal conductance phenotypes of *ztl-3* resembled the strong *ost1-3* phenotype.

A detached-leaf assay revealed that *ztl-3* and *ost1-3* leaves have significantly higher rates of water loss than WT leaves at ZT 8–9 (Figure 1E).

To further determine the role of ZTL in the drought response, we measured the water loss phenotype of *ztl-3* plants overexpressing *ZTL* under the control of the 35*S* CaMV promoter (*p35S::HA::ZTL*). There were no differences in the rates of water loss between WT plants and three independent T_3_ lines overexpressing ZTL in the *ztl*-3 background (Figure 1F); thus, *ZTL* could rescue the leaf water loss phenotype of *ztl-3*. This indicated that ZTL and OST1 are both needed for stomatal closure under stress.

We also analysed seed germination time (determined by radicle emergence), a developmental phase transition, to establish whether ZTL and OST1 showed shared effects across biological responses (Penfield and Hall, 2009). Both *ztl-3* and *ost1-3* mutants showed a similar significant delay in radicle emergence compared to WT seeds (Supplementary Figure S1).

### Circadian Clock Function Depends on ZTL in *Populus* Trees

**Figure 2:**
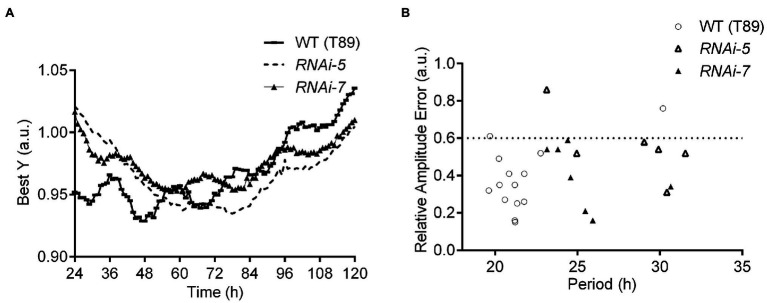
Delayed fluorescence (DF) in *Populus* leaves from WT (T89) trees and *PttZTL 1,2* RNAi lines assayed under continuous light. **(A)** Best fitted Y traces of DF rhythms in leaves in constant conditions following entrainment in LD 18:6 cycles. **(B)** Values of circadian period and relative amplitude errors (RAE) of individual leaves included in the experiment shown in **(A)**. RAE of rhythmic plants ≤0.6; period estimates are shown in Table 1.

Circadian components and ABA-signalling pathways are largely conserved between Arabidopsis and *Populus* (Ibáñez et al., 2010; Kozarewa et al., 2010; Cai et al., 2017; Yu et al., 2017; Tylewicz et al., 2018; Rigoulot et al., 2019). We hypothesised that ZTL’s roles in regulating the circadian clock and physiological responses to drought and ABA would also be conserved. The role of ZTL was examined in transgenic *Populus* trees in which expression of both *PttZTL1* and *PttZTL2* was downregulated by RNAi (*PttZTL1,2* RNAi lines). RNAi line 5, which had a strong reduction in *PttZTL* (Figures 2A,B; Supplementary Figure S2), leading to earlier growth cessation (Supplementary Figure S3), and RNAi line 7, which also showed a strong, significant reduction of *PttZTL* transcript (Figures 2A,B; Supplementary Figure S2), were selected for further analysis. Microarray expression profiles from short period (*lhy-10* RNAi) and WT *Populus* trees (Edwards et al., 2018) were examined for comparison (Supplementary Figure S4), together with an RT-qPCR analysis of time-series data from WT trees (Supplementary Figure S5). These data show *PttZTL1,2* expression appears disrupted in *lhy-10* trees in LD cycles (Supplementary Figure S4); thus, disruption of *PttLHY 1* and *PttLHY2*, expressed in the morning clock loop, apparently affects expression of *PttZTL1* and *2*, an evening gene, in *Populus* as in Arabidopsis. *PttZTL* expression is rhythmic in WT *Populus* leaves (Supplementary Figure S5).

Clock function was analysed in *PttZTL1,2* RNAi lines 5 and 7. Their circadian periods were measured in detached leaves using delayed fluorescence from photosystem II (Gould et al., 2009; Johansson et al., 2015). The normalised fluorescence traces (Best Y) showed both RNAi lines had significantly longer circadian periods than WT trees (Figure 2A; Table 1). The associated relative amplitude error (RAE) values indicate the level of rhythmicity associated with an individual leaf (Figure 2B); values ≥0.6 (dotted line) indicate arrhythmia. These results are in line with the downregulation of *PttZTL1,2* in RNAi lines 5 and 7 (Supplementary Figures S2A,B). Downregulation of *PttZTL1,2* expression resulted in a slower running but still strongly rhythmic circadian clock, as shown by the longer periods and low RAE values, indicating that the phenotypes of *PttZTL1,2* RNAi trees resembled those of Arabidopsis *ztl* mutants (Kevei et al., 2006).

**Table 1:** Free-running periods of delayed fluorescence in leaves of wild-type (WT; T89) *Populus* trees and *PttZTL1,2* RNAi lines 5 and 7 measured under continuous light.

Genotype	Period (h)	Error ± 1SE	Number of leaves (^$^Rhythmic/total)	Sidak’s *post-hoc* test (*α* < 0.05)
WT	21.1	±0.2	12/18	N/A
*RNAi-5*	29.2	±1.1	5/9	^****^
*RNAi-7*	25.4	±0.9	7/9	^***^

Populus trees were grown under LD 18:6 cycles; light intensity (equal parts blue and red light): 20 μmol m^−2^ s^−1^. Plants were transferred to continuous light (LL) at ZT 0 (dawn). Free-running rhythms (24–120 h after transfer to LL) were analysed using BRASS. ^$^Only rhythmic traces (relative amplitude error (RAE) ≤0.6) were included. Significance levels of the effects identified by one-way ANOVA (p < 0.0001) were determined using Sidak’s multiple comparisons post-hoc test; ^***^p < 0.001; ^****^p < 0.0001. N/A, not applicable.

Thus, these lines provided suitable material for further studies comparing ZTL-dependent function in ABA-related stress responses across plant species.

### ZTL’s Role in Stomatal Movements Is Conserved

**Figure 3:**
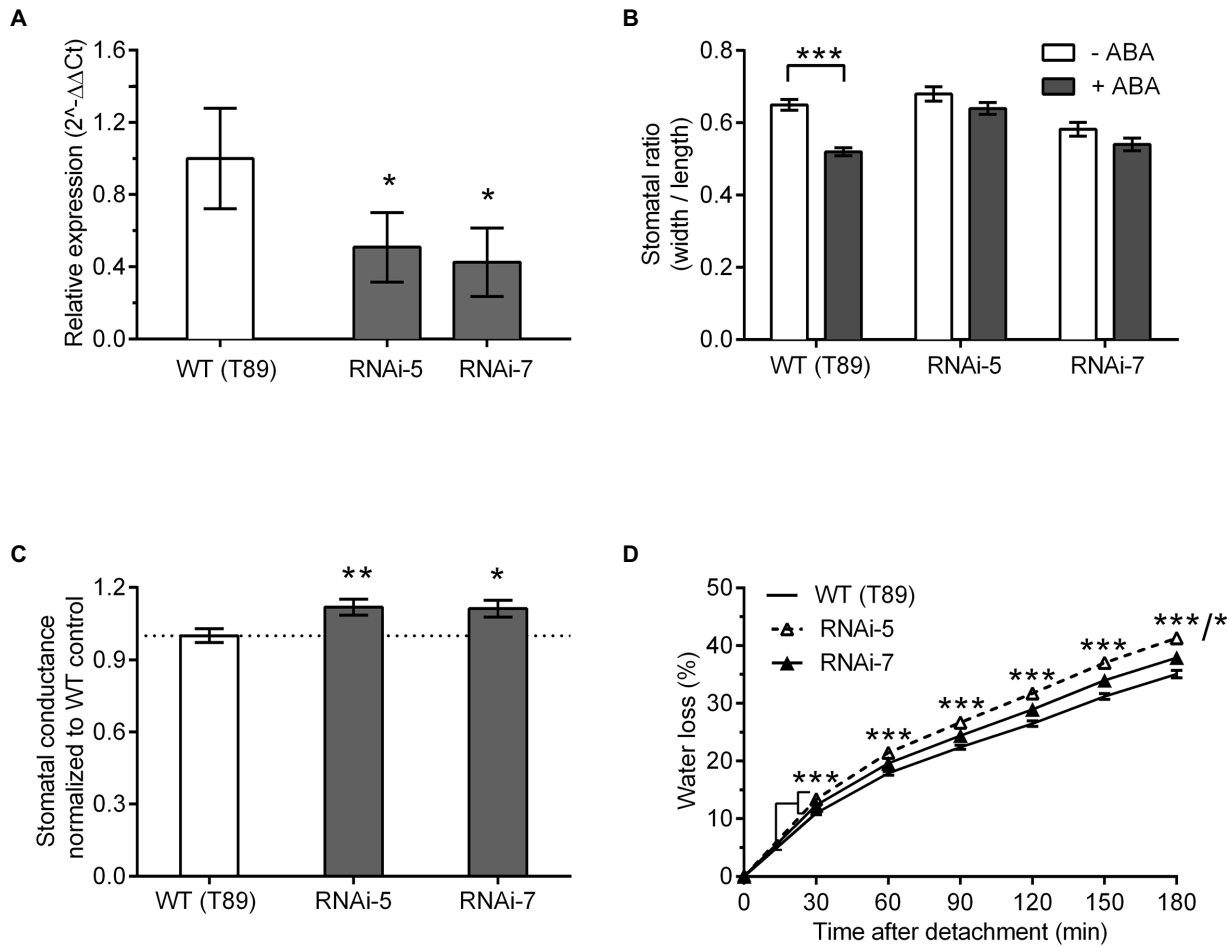
Effect of ZTL expression on stomatal phenotype in *Populus*. **(A)** Relative expression levels of *PttZTL* at ZT 8 in two *PttZTL 1,3* RNAi lines of *Populus*. Expression levels of *PttZTL* were normalised against expression of *EF1a* and the ratio was set at 1 in WT (T89) trees. Values are means ± SE of three or four biological replicates. **(B)** Effect of ABA treatment on stomatal ratios (width:length) in epidermal strips from wild-type and *PttZTL 1,2* RNAi lines measured at ZT 8–9 under CO_2_-free aeration. Data are means ± SE of three biological replicates, each containing 20 to 22 stomata. **(C)** Stomatal conductance (gs) in leaves from intact 6 to 9-week-old WT and *PttZTL 1,2* RNAi trees. Values are means ± SE of three biological replicates, each containing six to eight plants with three leaves/genotype. Conductance was normalised against the WT means. **(D)** Rates of water loss from detached leaves from 13-week-old *PttZTL* RNAi and wild-type trees. Values are means ± SE of three leaves per biological replicates, from six plants per genotype. In **(D)**, both RNAi lines differed from WT all time points: *** thus represents both the RNAi-5 vs. WT and RNAi-7 vs. WT comparisons at time points where RNAi lines differed from WT at the same level of significance. At 180 min, WT vs. RNAi line 5 differed from WT at *p* < 0.001 and RNAi line 7 differed from WT at *p* < 0.05; this is represented by ***/* on the Figure. **(A–D)** Differences between WT compared with both mutants are indicated where statistically significant at ^*^p < 0.05; ^**^p < 0.01; and ^***^p < 0.001 (Student’s *t*-test).

To investigate whether ABA-induced stomatal closure was conserved between Arabidopsis and *Populus*, we confirmed downregulation of ZTL at ZT 8–9 in *Populus* (i.e. at dusk, when ZTL levels peak and sensitivity to ABA are high in WT Arabidopsis) using an additional reference gene (*Ef1a*; Figure 3A). This confirmed that *PttZTL1* and *PttZTL2* were reduced to at least ~40% of WT levels in both lines at ZT 8–9. Further, we measured stomatal aperture in leaves from *Populus* trees grown *in vitro* (Figure 3B). Stomatal closure in response to ABA was reduced in RNAi lines 5 and 7, relative to WT plants. In addition, stomatal conductance in leaves from both *PttZTL1,2* RNAi lines was significantly higher than that of WT leaves (Figure 3C). These results resembled those from Arabidopsis *ztl* mutants (Figures 1A–D).

Next, we investigated water loss using detached-leaf assays. Both *PttZTL1,2* RNAi lines 5 and 7 showed significantly higher rates of water loss than WT plants (Figure 3D), similar to the Arabidopsis *ztl-3* mutant (Figures 1E,F). Stomatal density measurements revealed leaves from *PttZTL1,2* RNAi line 5 (135 stomata/mm^2^) differed significantly from WT leaves (141 stomata/mm^2^; Student’s *t*-test; *p* = 0.04; *n* = 3 biological replicates, each containing 120 to 144 measurements); however, there was no significant difference in stomatal density between WT leaves and leaves from *PttZTL1,2* RNAi line 7 (141 stomata/mm^2^; Student’s *t*-test *p* = 0.89; *n* = 3 biological replicates, each containing 120 to 144 measurements). These results indicated the increased water loss resulted from greater stomatal openness, not changed stomatal density. The lower sensitivity to ABA, increased stomatal aperture and conductance, and higher water loss observed in both *PttZTL1,2* RNAi lines (Figure 3) indicated they phenocopied Arabidopsis *ztl* mutants (Figure 1), supporting a conserved role for ZTL between the two species.

### Expression of ABA-Signalling Genes Is Impaired in Arabidopsis *ztl* Mutants

**Figure 4:**
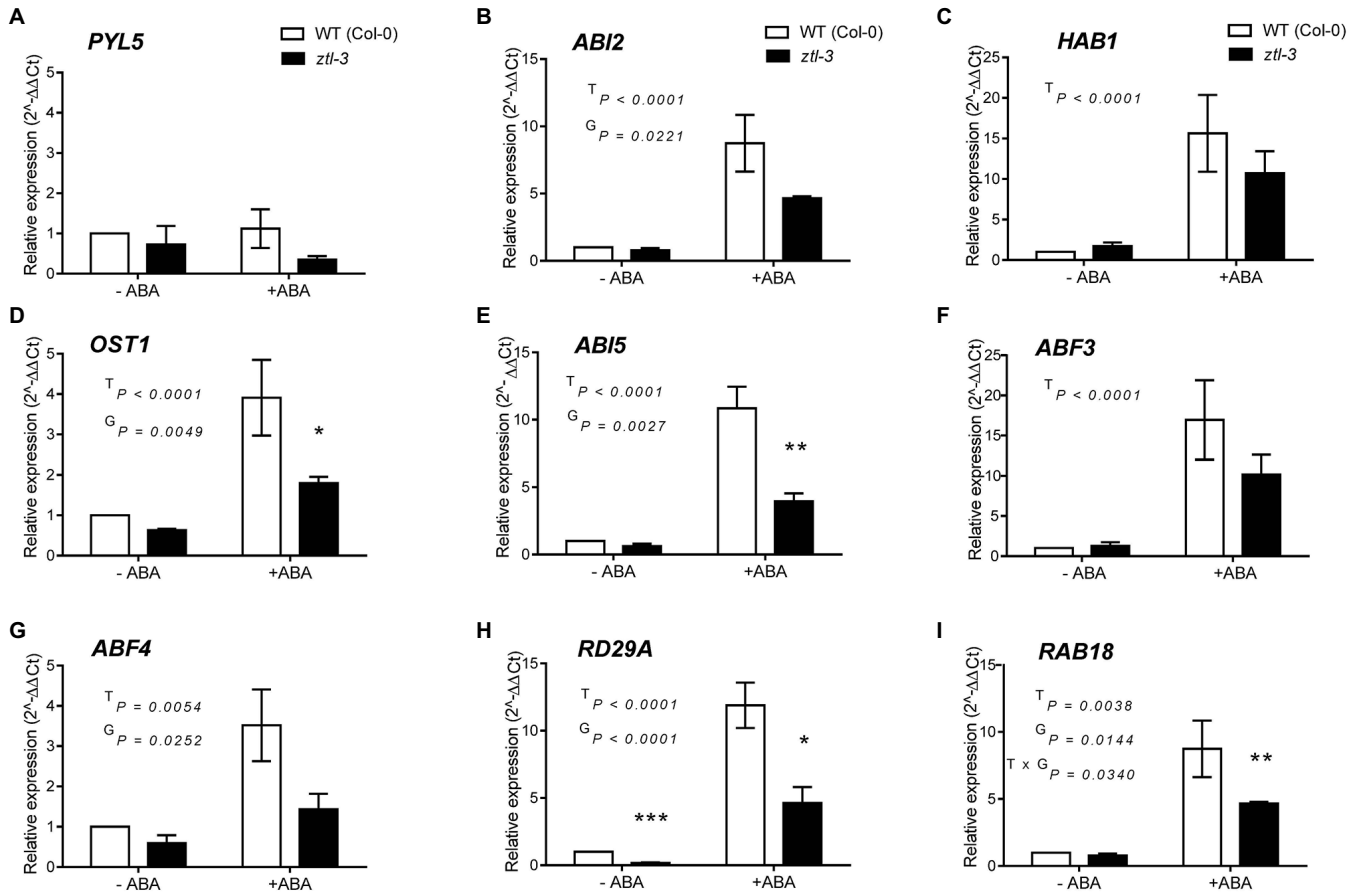
Effects of genotype and ABA on expression of early and late ABA-signalling components and ABA-responsive genes in Arabidopsis. **(A)** ABA reception gene *PYL5*. **(B–G)** Early and progressing ABA-signalling genes. **(B)** *ABI2*. **(C)** *HAB1*. **(D)** *OST1*. **(E)** *ABI5*. **(F)** *ABF3*. **(G)** *ABF4*. **(H,I)** Late responsive ABA-signalling genes. **(H)** *RD29A*. **(I)** *RAB18*. Gene expression was measured in samples collected at ZT 8 from WT (Col-0) and *ztl-3* plants treated with ABA or ethanol (control). Values are the means ± SE of pooled seedlings from three biological replicates, each containing two technical replicates. Expression levels were normalised against expression of *EF1a* and the ratio was set at 1 in untreated WT (Col-0). Results were analysed by two-way ANOVA to determine the effects of treatment (T), genotype **(G)** and the T × G interaction. Significance levels of the effects were determined using Sidak’s multiple comparisons *post-hoc* test; ^*^p < 0.05; ^**^p < 0.01; and ^***^p < 0.001.

Given stomatal closure in response to ABA was impaired in *ztl* mutants (Figures 1A–C) and the similar changes in stomatal regulation in Arabidopsis and *Populus* trees with reduced ZTL function (Figures 2, 3), we evaluated whether ZTL modulated expression of early and late ABA-signalling components and ABA-responsive genes in Arabidopsis. We analysed expression of key ABA response and signalling genes in *ztl-3* and WT plants treated with ABA for 3 h (Figure 4). *PYL5* was selected as a representative of an ABA reception gene, *ABI2, HAB1, OST1*, *ABI5*, *ABF3* and *ABF4* as examples of early and progressing ABA-signalling genes, and *RAB18* and *RD29A* as late ABA-responsive genes (Santiago et al., 2009; Raghavendra et al., 2010; Gonzalez-Guzman et al., 2012).

Analysis by two-way ANOVA indicated there were no treatment (T), genotype (G) and treatment × genotype (T × G) effects on *PYL5* expression (Figure 4A). Analysis of expression of ABI2 and HAB1 (Figures 4B,C), two negative regulators of ABA signalling (Saez et al., 2004, 2006), revealed that genotype had a significant effect on *ABI2*, with a significant reduction in transcript level in *ztl-3* (Figure 4B), but not on *HAB1*, which only showed a treatment effect (Figure 4C). Both treatment and genotype had significant effects on *OST1*, expression of which was significantly reduced in *ztl-3* relative to wild-type plants (Figure 4D). Expression of the later ABA-signalling gene *ABI5* was severely diminished in response to ABA in *ztl-3* plants, with significant effects of both treatment and genotype, as well as a treatment × genotype interaction (Figure 4E). Only treatment affected *ABF3* expression (Figure 4F), but both treatment and genotype affected expression of *ABF4* (Figure 4G). Expression of another ABA-responsive gene, *RAD29A*, showed significant treatment and genotype effects (Figure 4H). Both treatment and genotype had significant effects on expression of *RAB18* (Figure 4I), and these factors interacted to produce a further significant effect. All these data show that ZTL was required for ABA sensitivity and significantly promoted the expression of major ABA-signalling components (Table 2).

**Table 2:** Summary of *post-hoc* analysis using Sidak’s multiple comparisons test of RT-qPCR analysis of gene expression in Arabidopsis wild-type (Col-0) and *ztl-3* plants ± ABA (Figure 4).

Gene	Treatment	Mean diff. (WT – *ztl-3*)	95% CI of diff.	Significance level *α* < 0.05
*PYL5*	−ABA	1.040	−1.605 to 3.685	ns
	+ABA	1.294	−1.351 to 3.938	ns
*ABI2*	−ABA	0.3944	−0.4455 to 1.234	ns
	+ABA	0.832	−0.007819 to 1.672	ns
*HAB1*	−ABA	−0.6830	−2.069 to 0.7031	ns
	+ABA	0.4794	−0.9068 to 1.866	ns
*OST1*	−ABA	0.6713	−0.1823 to 1.525	ns
	+ABA	1.024	0.1703 to 1.878	Yes, *
*ABI5*	−ABA	0.8129	−0.2108 to 1.837	ns
	+ABA	1.449	0.4254 to 2.473	Yes, **
*ABF3*	−ABA	−0.1650	−1.707 to 1.377	ns
	+ABA	0.6919	−0.8497 to 2.233	ns
*ABF4*	−ABA	0.9553	−0.6202 to 2.531	ns
	+ABA	1.276	−0.2995 to 2.852	ns
*RD29A*	−ABA	2.567	1.498 to 3.636	Yes, ***
	+ABA	1.440	0.3710 to 2.509	Yes, *
*RAB18*	−ABA	0.1204	−0.7153 to 0.9561	ns
	+ABA	1.221	0.3849 to 2.056	Yes, **

Statistical significances indicated ^*^p < 0.05; ^**^p < 0.01; and ^***^p < 0.001 (post-hoc analysis).

### ZTL Interacts With OST1 in Plant Cells

**Figure 5:**
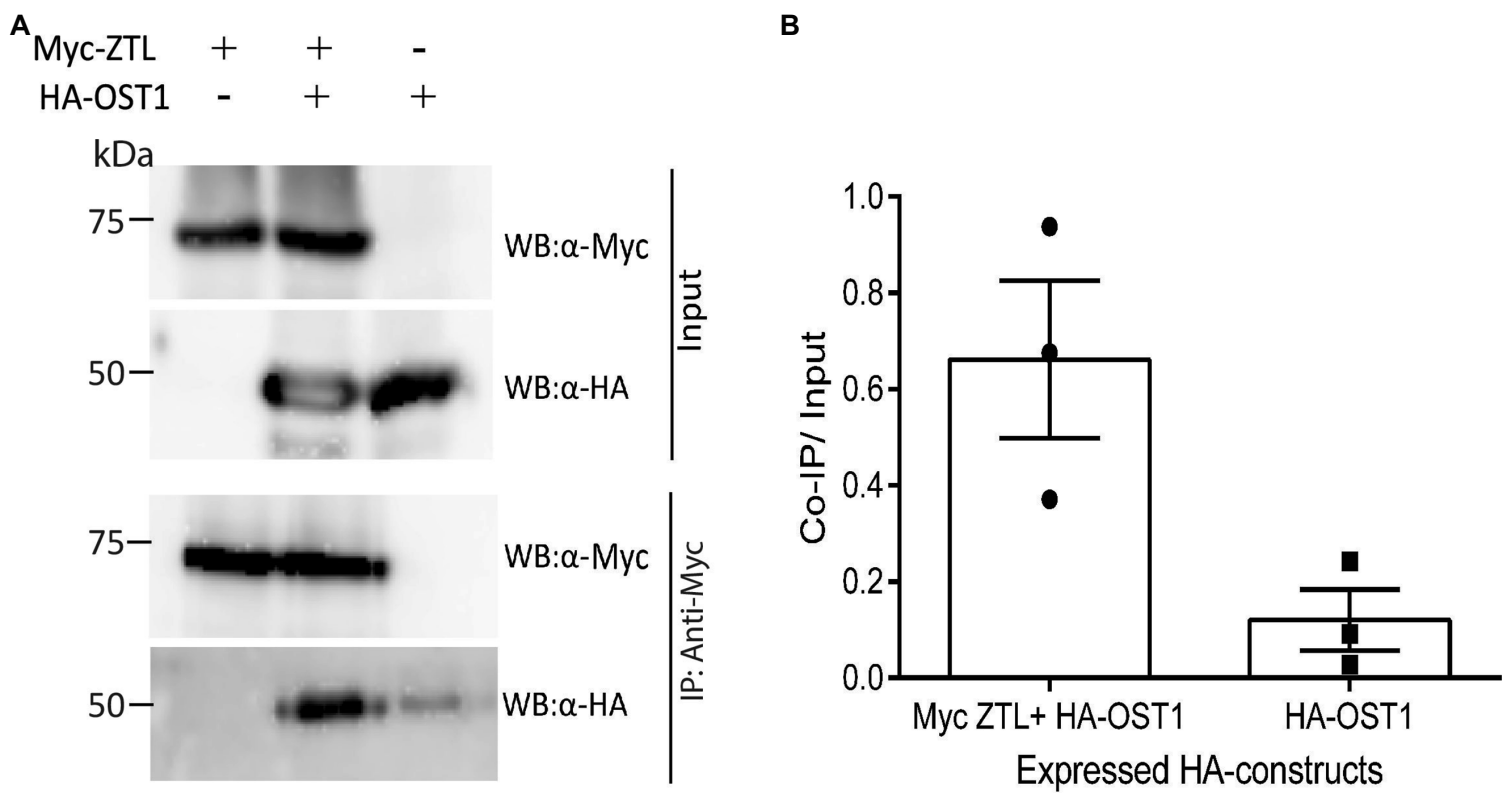
Coexpression of ZTL and OST1 in plant cells indicates a strong interaction between the proteins. **(A)** Representative co-immunoprecipitation assay showing *in vivo* interaction between ZTL and OST1. Tagged versions of ZTL and OST1 proteins were expressed in Arabidopsis protoplasts, singly or in combination. Proteins were immunoprecipitated using mouse anti-Myc antibody (IP: α-Myc) and subsequently analysed by Western blotting with an anti-HA-POD antibody (WB: α-HA) and anti-c-Myc chicken antibody (WB: α-Myc). The experiment was repeated three times in different Western blots and produced similar results. **(B)** Mean values (*n* = 3) ± SEM of the ratio of Co-IP signal to sample signal from the input reaction (40% of sample used in Co-IP) of the three different co-immunoprecipitation assays described in **(A)**. The individual results from each experiment are shown on the plot: Filled circles (left-hand side): Myc-OST1 + HA-ZTL; filled squares (right-hand side): HA-ZTL.

Given the function of ZTL in stomata closure and in ABA-induced gene expression, we tested if it has the capacity to interact with OST1 when expressed in plant cells. Co-immunoprecipitation (Co-IP) assays following expression of tagged ZTL and OST1 proteins in protoplasts revealed an interaction between ZTL and OST1 (Figure 5A). The ratios of signal from Co-IP bands to input, measured from three experiments, showed a strong signal from HA-OST1, when it was coexpressed with Myc-OST1 (Figure 5B). Co-IP of the known interactors, ZTL and TOC1, showed a similar result (Supplementary Figures S6A,B; Johansson et al., 2011).

**Figure 6:**
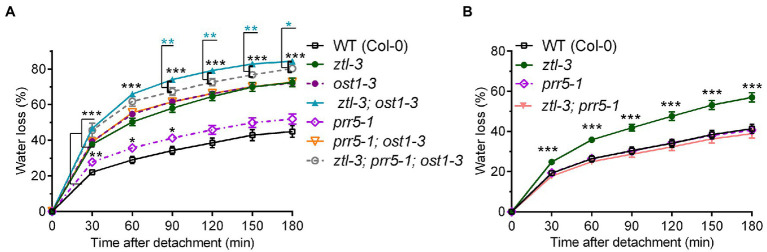
ZTL and OST1 act synergistically to promote stomatal closure, and PRR5 partially blocks ABA signalling in the absence of both ZTL and OST1. **(A, B)** Water loss from detached rosette leaves of 3-week-old WT (Col-0) Arabidopsis and single, double and triple mutants carrying different combinations of alleles at the *ZTL* (*ztl-3*), *PRR5* (*prr5-1*) and *OST1* (*ost1-3*) loci. Values are means ± SE of two to three biological replicates, each containing one leaf from six to eight plants of each genotype. In **(A)**, significant differences between the *ztl-3 and ost1-3* double mutant and the *ztl-3, prr5-1 and ost1-3* triple mutant are shown by cyan-coloured asterisks. As all the mutants differed from WT at *p* < 0.001 (Student’s *t*-test) at all time points, all the pairwise mutant vs. WT comparisons represented by three black asterisks for simplicity. In **(B)**, pairwise comparisons found that *ztl-3* differed significantly from WT but *prr5-1* and the *prr5-1 and ztl-3* double mutant did not. The result for *ztl-3 vs* WT (Student’s *t*-test) is represented by three black asterisks. Statistical levels in **(A)** and **(B)**: ^*^p < 0.05; ^**^p < 0.01; and ^***^p < 0.001.

The effect of ZTL on OST1 stability was investigated using mesophyll protoplasts obtained from the triple mutant *ztl-4, fkf1-2 and lkp2-1* (Supplementary Figure S7). This mutant lacks all three members of the ZTL F-box protein family [ZTL, FLAVIN-BINDING, KELCH REPEAT, F BOX 1 (FKF1) and LOV KELCH PROTEIN 2 (LKP2)] (Baudry et al., 2010). ZTL-like activities were diminished in this background, as expected. No ZTL-dependent degradation of OST1 was detected in protoplasts transfected with OST1 and ZTL, regardless of whether the proteasome inhibitor MG132 was present (Supplementary Figures S7A,B, S8). The effect of proteasome inhibition was also tested using TOC1, and in contrast, coexpression of TOC1 and ZTL led to lower TOC1 expression at time 0 (Supplementary Figures S7C,D). Although the effect on TOC1 in blue light was not entirely consistent with the current model, wherein ZTL acts to degrade TOC1 in the dark (Más et al., 2003), the lack of activity of the ZTL family members FKF1 and LKP2 in the triple mutant protoplasts affected TOC1 expression (Supplementary Figures S7C,D), consistent with the results of Baudry et al. (2010). OST1 stability, on the other hand, showed no effect of ZTL coexpression or proteasome inhibition (Figures 6A,B), which supports our conclusion that ZTL does not regulate OST1 protein levels. OST1 stability was not negatively affected in protoplast suspension cultures, regardless of the presence of ZTL, light or CHX (Supplementary Figure S8).

### Both ZTL and PRR5 Interact With OST1 to Modulate Stomatal Regulation

**Figure 7:**
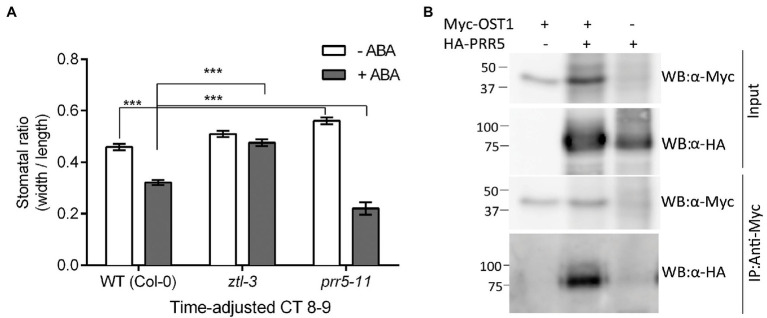
The circadian clock protein PRR5 interacts with ZTL and OST1 to regulate stomatal responses in Arabidopsis. **(A)** *ztl-3* and *prr5-11* mutants show opposite stomatal aperture phenotypes in response to ABA. Time of ABA treatment was adjusted to an equivalent point in the circadian cycle to accommodate the difference in period (*τ*) between *ztl-3* (*τ* = ~ 28 h), *prr5-11* (*τ* = ~ 23 h) and wild-type (*τ* = 24 h) plants. Values are means ± SE of three biological replicates, each containing 20 stomata/genotype. (^***^p < 0.001; Student’s *t*-test). **(B)** PRR5 and OST1 interact *in vivo*. Co-immunoprecipitation assay showing an interaction between PRR5 and OST1 in Arabidopsis protoplasts. Tagged versions of PRR5 and OST1 proteins were expressed in Arabidopsis protoplasts. Proteins were immunoprecipitated using mouse anti-Myc antibody (IP: α-Myc) and subsequently analysed by Western blotting with an anti-HA-POD antibody (WB: α-HA) and anti-c-Myc chicken antibody (WB: α-Myc).

The genetic data suggested that ZTL and OST1 both severely and similarly affected stomatal closure in response to ABA (Figure 1E). In order to determine if additional factors were involved, we investigated the role of PRR5, a ZTL substrate that affects ABA metabolism and induced responses (Yang et al., 2021). We adjusted ABA treatment time to match the circadian period of each genotype (*ztl-3*: 28 h; *prr5-11*: 23 h and WT: 24 h) and scored the stomatal ratio at Circadian Time (CT) 8–9 for each genotype to confirm that the phenotypic change resulted from ABA treatment rather than circadian mistiming. Under these conditions, the stomata of *ztl-3* mutants failed to close following ABA treatment, while stomata of *prr5-11* closed more readily (Figure 7A). The responses of both mutants to ABA differed significantly from WT plants, albeit in opposite directions, consistent with the changes in endogenous period.

Given that ZTL mediates proteasomal degradation and several *ztl* mutants show alterations in PRR5 protein stability and other alterations that may affect PRR5 function (Kiba et al., 2007; Fujiwara et al., 2008), we hypothesised that altered ZTL-dependent regulation, for instance stabilisation of PRR5 in the *ztl-3* mutant, might increase stomatal opening. Consistent with this, PRR5 appeared to be required for normal stomatal responses. Although *prr5-1* mutants remained sensitive to ABA and showed stomatal closure in response to ABA treatment, in the absence of exogenous ABA, their stomata were more open than those of WT plants (Figure 7A). This may result from an increase in ZTL levels in *prr5-1* in the absence of ABA. We found a clear interaction between PRR5 and OST1 in plant cells (Figure 7B).

To understand how OST1, ZTL and PRR5 interacted to regulate stomata (i.e. responsiveness to ABA), we performed water loss assays using detached leaves from a series of single, double and triple mutants carrying different combinations of the *ztl-3*, *ost1-3* and *prr5-1* alleles to determine their physiological responses to drought (Figure 6A). The *ztl-3* and *ost1-3* single mutants had a similar phenotype that differed from that of WT plants, consistent with previous data implicating both ZTL and OST1 in stomatal regulation; moreover, each single mutant differed significantly from the double mutant, *ztl-3 and ost1-3*. The increased severity of the loss-of-function phenotype in the *ztl-3 and ost1-3* double mutant indicated both ZTL and OST1 were required for stomatal control and potentially acted additively to each other.

Leaves from *prr5-1* single mutants showed rates of water loss similar to WT with slight deviations (Figures 6A,B). The double mutant *ztl-3 and prr5-1* behaved as WT (Figure 6B). The double mutant *prr5-1 and ost1-3* behaved like *ost1-3* (Figure 6A). The *ztl-3 and ost1-3* double mutant had the strongest water loss phenotype, indicating that retaining PRR5 in the absence of OST1 and ZTL partially blocked regulation of stomatal aperture by ABA. The *ztl-3, prr5-1 and ost1-3* triple mutant showed a significantly lower rate of water loss than the *ztl-3 and ost1-3* double mutant between 90 and 150 min, and again at 180 min, which is consistent with the suggestion that PRR5 is involved in ABA signalling in the absence of ZTL and OST1.

These results taken together suggest that inhibition of the ABA-signalling pathway by PRR5 is opposed by ZTL and OST1. The *ztl-3, prr5-1 and ost1-3* triple mutant had a significantly stronger water loss phenotype than the *ost1-3* single mutant, indicating that ZTL can oppose the inhibitory effect of PRR5 in the absence of OST1. By contrast, the *prr5-1 and ost1-3* double mutant and the *ost1-3* and *ztl-3* single mutants all showed very similar phenotypes that were less extreme than the *ztl-3, prr5-1 and ost1-3* triple mutant (Figure 6A), indicating the importance of the interactions between ZTL, OST1 and PRR5.

## Discussion

### Water Loss Is Regulated by Both Clock and ABA-Signalling Pathways

In Arabidopsis, light and the circadian clock act *via* ZTL to regulate the daily pattern of stomatal opening (Somers et al., 1998; Salomé et al., 2002; Dodd et al., 2004). Many metabolic processes and enzymes associated with photosynthesis are under circadian control (Dodd et al., 2014). We show that ZTL was also required for circadian clock function (Figure 2; Table 1) in *Populus*, and ZTL regulated stomatal closure in both Arabidopsis and *Populus* (Figures 1, 3). In Arabidopsis, both OST1- and ABA-dependent gene expression and integration of ABA-signalling require ZTL (Figure 4; Table 2). ZTL regulated the physiological responses of guard cells to ABA and drought by acting in synergy with OST1 (Figures 1, 5, 6).

OST1 is a central component of ABA signalling and stomatal closure. The increased rate of water loss from Arabidopsis *ztl* mutants as well as their impaired stomatal regulation in response to ABA (Figure 1) suggests a requirement of ZTL for normal *OST1* function. The similar phenotypes observed in *Populus* trees with reduced ZTL function (Figure 3) indicates a similar mechanism operates in *Populus*.

### OST1 Interacts With the Clock Proteins ZTL and PRR5

We showed that ZTL bound directly to OST1 in plant cells. Loss-of-function *prr5-11* mutants showed an opposite stomatal phenotype to *ztl-3*, a null mutant (Somers et al., 2004) that does not produce mRNA or protein (Figure 7A). The water loss phenotype of the *prr5-1* mutant (a low level of water loss comparable to WT) was as expected, given the *prr5-11* mutant showed high levels of stomatal closure in the presence of ABA (Figure 7A). In contrast, the stomata of the *ztl-3* and *ost1-3* single mutants remained more open in response to ABA (Figure 7A), which matched the high levels of water loss shown by these mutants (Figures 6A,B). The genetic data confirmed that a triple mutant that combined the loss-of-function *prr5-1* allele with the *ztl-3* and *ost1-3* alleles had a lower rate of water loss than a *ztl-3 and ost1-3* double mutant but did not completely phenocopy it (Figure 6A). This may result from TOC1 accumulation, in the triple mutant as TOssC1 is a substrate of ZTL that affects ABA responses (Legnaioli et al., 2009); moreover, overexpression of TOC1 slightly increases stomatal apertures and reduces water use efficiency (Legnaioli et al., 2009; Simon et al., 2020).

We hypothesised that an increased level of PRR5 in the absence of ZTL would exacerbate the water loss phenotype. Thus, we carried out physiological, biochemical and genetic assays to probe PRR5 function in stomatal closure, rate of water loss and interaction with OST1 (Figures 6, 7). Direct interactions between ZTL and OST1 (Figure 5), and between PRR5 and OST1 (Figure 7B), occurred in plant cells. In addition, the level of PRR5 affected stomatal aperture (Figure 7A). Removing both OST1 and ZTL function (*ztl-3 and ost1-3* double mutant) produced a strong water loss phenotype (Figure 6A); however, a triple mutant (*ztl-1, prr5-1 and ost1*-3) showed a less extreme water loss phenotype (Figure 6A), indicating that, in the absence of ZTL and OST1, PRR5 partially blocked the ABA pathways controlling stomatal aperture. The interactions between OST1, ZTL and PRR5 are thus essential for ABA-signalling and water regulation under stressful conditions. ABA treatment may produce a change in circadian period which is dependent on genotype and thus, the effect of ZTL on ABA-signalling gene expression may result from a phase shift (Legnaioli et al., 2009; Liu et al., 2013), although, in our hands, treatment with 20 μM ABA did not significantly alter the phase in the first 24 h (Supplementary Figure S9) or period of WT and *ztl-21* seedlings (Supplementary Figure S9; Supplementary Tables 1, 2).

These findings suggest that both ZTL and PRR5 interact with OST1 to regulate stomata and ABA-dependent responses. As the loss of PRR5 did not influence the *ost1-3* water loss phenotype (Figure 6A), PRR5 appears to act upstream of OST1. In contrast, the loss of PRR5 strongly influenced the water loss phenotype of *ztl-3* mutants (Figure 6B). PRR5 acting downstream of ZTL but upstream of OST1 would also explain the phenotype of the *ztl-3, prr5-1 and ost1-3* triple mutant. These physical and genetic interactions provide a biochemical framework linking ZTL directly to ABA-regulated components. ZTL thus acts with OST1 as a circadian short-cut to help phosphorylate and/or degrade PRR5 enabling osmotic regulation of guard cells and connecting the clock with responses essential for water conservation.

### The Roles of ZTL and OST1 in Regulating Water Loss Are Conserved Across Plant Species

There is a significant overlap between ABA and cold signalling pathways and control by the circadian clock (Eriksson and Webb, 2011); for instance, the circadian MYB-transcription factors CCA1 and LHY contribute to cold responses in both Arabidopsis and *Populus* sp. (Espinoza et al., 2010; Ibáñez et al., 2010; Dong et al., 2011). The circadian clock regulates ABA signalling *via* transcriptional changes induced by TOC1 (Legnaioli et al., 2009; Huang et al., 2012). Mutations at the circadian clock-associated *EARLY BIRD/NFX1-LIKE 2* locus also increase resistance to salt and drought stress (Lisso et al., 2006), as well as inducing hypersensitive responses to ABA (Lisso et al., 2012). Genome-wide analysis of Arabidopsis recently showed that LHY controlled expression of ABA biosynthesis and receptor genes, as well as other aspects of signalling (Adams et al., 2018). We investigated several of these genes, including *ABI2*, *OST1*, *ABI5, ABF4* and *RD29A*, and found ZTL affected their expression (Figure 4).

The receptors regulating ABA responses have increased in number since plants first colonised dry land (Umezawa et al., 2010) in response to the need to manage water status and detect and respond to heat and drought stresses. Such stresses are largely managed by controlling stomata. Stomata provide a means of CO_2_ entry, thus enabling photosynthesis, and also of controlling water loss through transpiration. They thus are critical regulators of plant growth.

Stomatal conductance is a crucial trait affecting water status and photosynthetic capacity that directly impacts biomass accumulation of trees. Stomatal conductance is under diurnal regulation in field-grown *Eucalyptus sp*., and thus likely controlled by the clock (Resco de Dios et al., 2013). We found previously that *Eucalyptus sp*. exhibit robust circadian rhythms under constant conditions (Johansson et al., 2015), and our present data suggest that ZTL acts in similar ways in both Arabidopsis and *Populus* to link the clock to stomatal control. Thus, the important roles of ZTL and OST1 in controlling stomata is conserved across species.

Our work highlights plants’ dependence on the circadian clock to respond to drought stress. Studies in *Populus balsamifera (Pb)* suggest that *PbZTL2* is under local climatic selection, as are ABA-related signalling components, *PbGIs* and additional clock-associated genes (Keller et al., 2017). In addition, in *Populus trichocarpa*, *PtPRR5*, *PtPRR7* and other clock genes are associated with biomass, phenology and physiological traits in Genome-Wide Association Studies (GWAS) and appear to have undergone selection (McKown et al., 2014); for example, *PtPRR5* is part of an adaptive introgression of genes on Chromosome 15 transferred from *P. balsamifera* into *P. trichocarpa* (Suarez-Gonzalez et al., 2016).

OST1 is a kinase and thus a target for both phosphorylation and ubiquitination (Kim et al., 2013). The partnership between ZTL and OST1 underlies changes in ubiquitination and/or phosphorylation. Their associations with PRR5 may facilitate localisation of PRR5 to the nucleus and its interactions with, for instance, ABI5. The circadian clock PRR proteins are expressed sequentially between dawn and dusk in the order PRR9, PRR7, PRR5 and TOC1/PRR1. This may provide a set of ‘cogs’ enabling interactions with OST1, with or without ZTL, to be integrated into circadian clock and ABA-signalling pathways and respond to abiotic stresses occurring at different times across the day. Other circadian genes including *PRR7* influence ABA signalling (Liu et al., 2013). Future efforts to resolve the underlying mechanisms controlling stomatal regulation and stress tolerance will benefit from consideration of the interaction between OST1, ZTL and the PRRs. Further studies in both Arabidopsis and *Populus* will determine the detailed mechanisms controlling diel stomatal closure and ABA-signalling responses.

## Data Availability Statement

The original contributions presented in the study are included in the article/Supplementary Material, further questions can be directed to the corresponding author.

## Author Contributions

AARW and MEE conceived the research. MJu, MJo, IK, LB, AARW, MI-N, and MEE designed the experiments. MJu, AM, MJo, CI, IK, JS, NT, and MEE carried out the experiments and analysed the data. All authors interpreted the results and contributed to writing the manuscript.

## Funding

This research was funded by Carl Trygger Foundation for Scientific Research, the Kempe Foundations, the Swedish Governmental Agency for Innovation Systems (VINNOVA), the Swedish Research Council (VR), the Swedish Research Council Formas, Stiftelsen Nils and Dorthi Troëdsson Forskningsfond, Trees and Crops 4 the Future, Knut and Alice Wallenberg Foundation, and the Berzelii Centre for forest biotechnology. MI-N as VINNMER fellow was funded by VINNOVA. ME as VINNMER Marie Curie International Qualification Fellow was funded by VINNOVA and European Union and supported by an Umeå University career grant and also received support from Churchill College, Cambridge University, Cambridge, United Kingdom.

## Conflict of Interest

MEE is a member and CEO of the holding company Woodheads AB, a part-owner of SweTree Technologies (STT), which played no part in this work and she is also a board member of STT.

The remaining authors declare that the research was conducted in the absence of any commercial or financial relationships that could be construed as a potential conflict of interest.

## Publisher’s Note

All claims expressed in this article are solely those of the authors and do not necessarily represent those of their affiliated organizations, or those of the publisher, the editors and the reviewers. Any product that may be evaluated in this article, or claim that may be made by its manufacturer, is not guaranteed or endorsed by the publisher.

## Footnotes

^1^ http://signal.salk.edu

^2^ https://popgenie.org/

^3^ www.Arabidopsis.org/abrc/
