# Supplementary material for: ZEITLUPE Promotes ABA-Induced Stomatal Closure in Arabidopsis and Populus
Source: Front Plant Sci. 2022 Mar 2;13:829121. doi: 10.3389/fpls.2022.829121 (PMC8924544; doi:10.3389/fpls.2022.829121)
Supplement: Supplementary file 1 [file Data_Sheet_1.PDF]

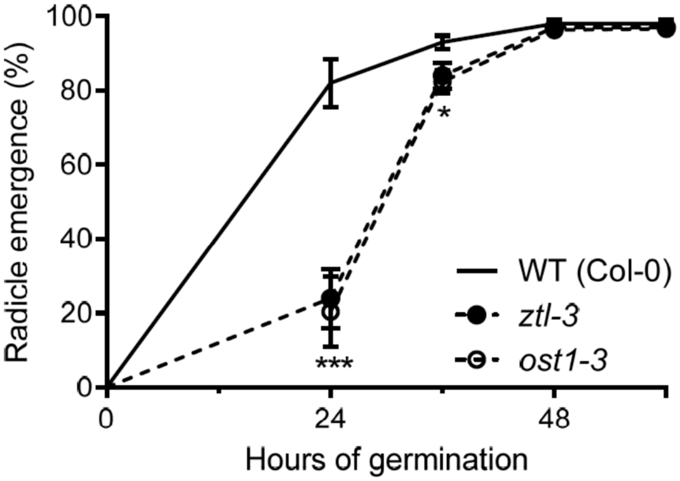

Supplementary Figure 1. Radicle emergence in *ztl-3, ost1-3* and WT (Col-0) seeds. The percentage of seeds showing radicle emergence was scored at 12 h intervals, starting at 24 h. The experiment was performed twice using seeds from separate batches with similar results. Values are means ± SE; n = 5 (60 seeds/genotype per replicate). WT seeds differed significantly from both *ztl-3* and *ost1-3* seeds by Student's *t*-test at 24 h ***: $P < 0.001$) and at 36 h *: $P < 0.01$. In each case the asterisks indicate both WT *vs ztl-3* and WT *vs ost1-3* comparisons.

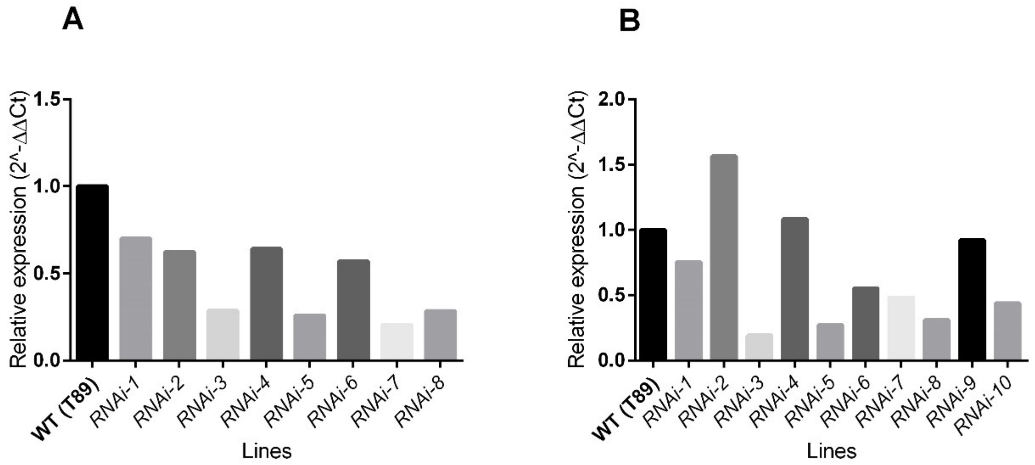

Supplementary Figure 2. Levels of *PttZTL1* and *PttZTL2* (combined*; PttZTL1, 2*) expression in independent *Populus* RNAi lines and WT (T89) *Populus* trees. (**A**) ZTL expression at ZT 12 in three leaves per line sampled from internodes 8, 9 and 10. (**B**) ZTL expression in pooled samples consisting of leaf 10 collected at five timepoints (every 4 h) over 24 h. Leaves were collected from 8 to 9-week-old plants grown under LD 18:6 in a greenhouse. Expression of *PttZTL* was determined using RT-qPCR and normalised against 18 S RNA expression in the same sample. RT-qPCR was performed in (**A**) on one biological pool and in (**B**) on three biological pools; in each case, with three technical replicates per pool of sampled leaves. The level of expression in WT plants WT was set to 1 in (**A, B**) and expression in the *PttZTL1, 2* RNAi lines was compared with this. The gene-specific primers for *PttZTL1, 2* and 18S rRNA have been previously published (Kozarewa et al., 2010) and their sequences are included in the Supplementary material.

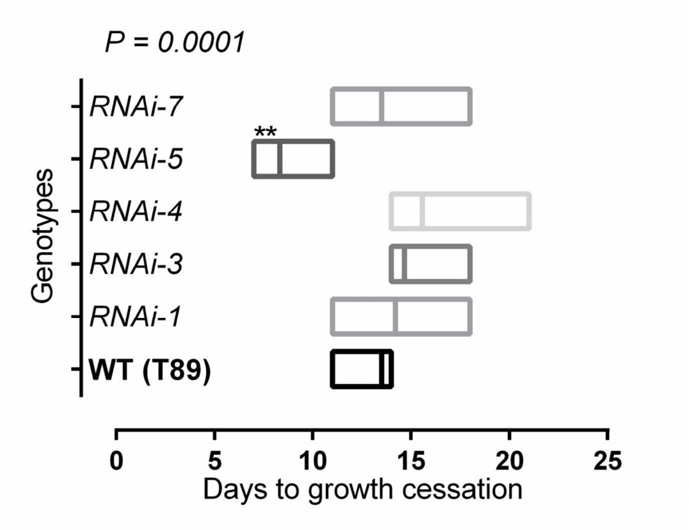

Supplementary Figure 3. Growth cessation of *PttZTL1,2* RNAi lines and WT (T89) *Populus* trees. Trees were transferred from a growth-inducing long day photoperiod (LD 18:6) to a growth cessation-inducing short photoperiod (LD 15:9); the temperature at both photoperiods was constant 18 °C. Four to seven biological replicates per line were analysed. One-way ANOVA revealed significant differences between genotypes and a significant response to short days ($P = 0.0001$). Sidak's *post hoc* test showed *PttZTL1,2* RNAi line 5 was significantly more sensitive than WT to daylength shortening; **: $P < 0.01$.

A

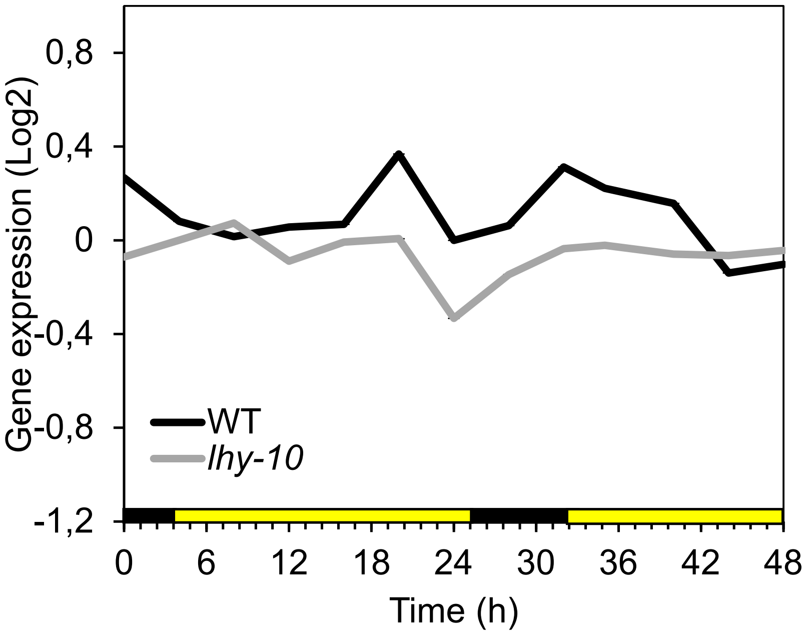

B

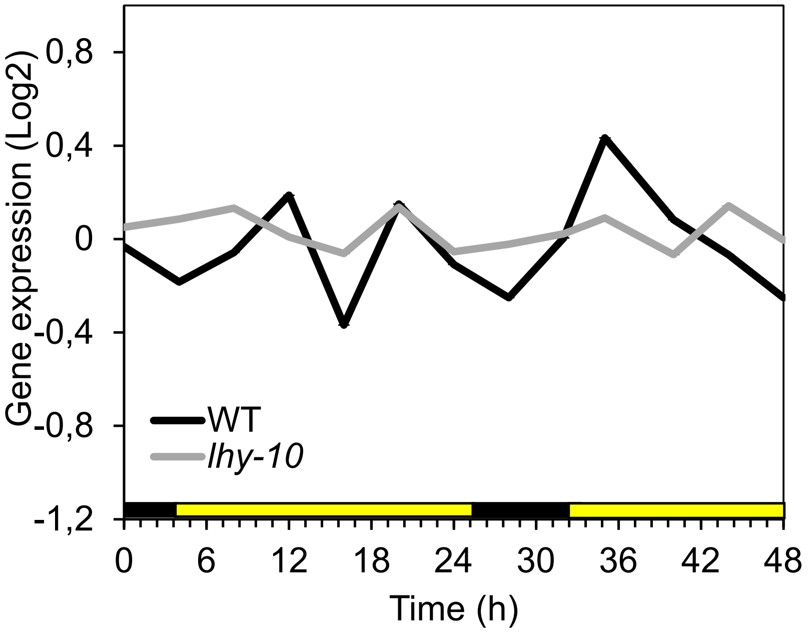

Supplementary Figure 4. Expression of *PttZTL1* and *PttZTL2* in *lhy-10* RNAi and WT (T89) *Populus* trees. (**A**) Affymetrix probe set PtpAffx.2892.2.S1_at, which matches *PttZTL1* gene model Potri.018G090800. (**B**) Affymetrix probe set PtpAffx.2892.2.S1_a_at*,* which matches gene models *PttZTL1* and *PttZTL2*Potri.006G166300 and Potri.018G090800 (Keller et al., 2018). Data from Edwards *et al.,* (2018). Trees were maintained under LD 18:6. Sampling started 3 hrs before dawn at ZT 21 (experimental time '0').

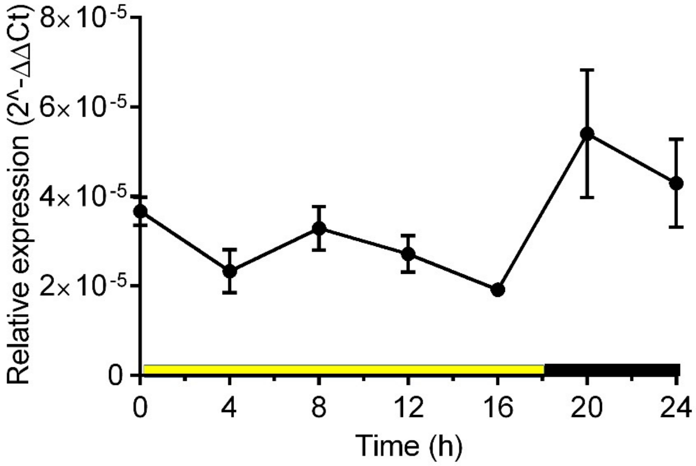

Supplementary Figure 5. Expression of *PttZTL1,2* in WT (T89) *Populus* leaves. Trees were maintained under LD 18:6 at 18 °C. *PttZTL1,2* expression was measured in pools of leaves of internodes 8, 9 and 10 sampled every 4 h between dawn and dawn using RT-qPCR. A green safe-light was used for collections during the dark period. Expression of *PttZTL1,2* was normalised against expression of 18S rRNA in the same sample, as described previously (Kozarewa et al., 2010; Supplementary material). Error bars are means ± SE of 3 technical replicates.

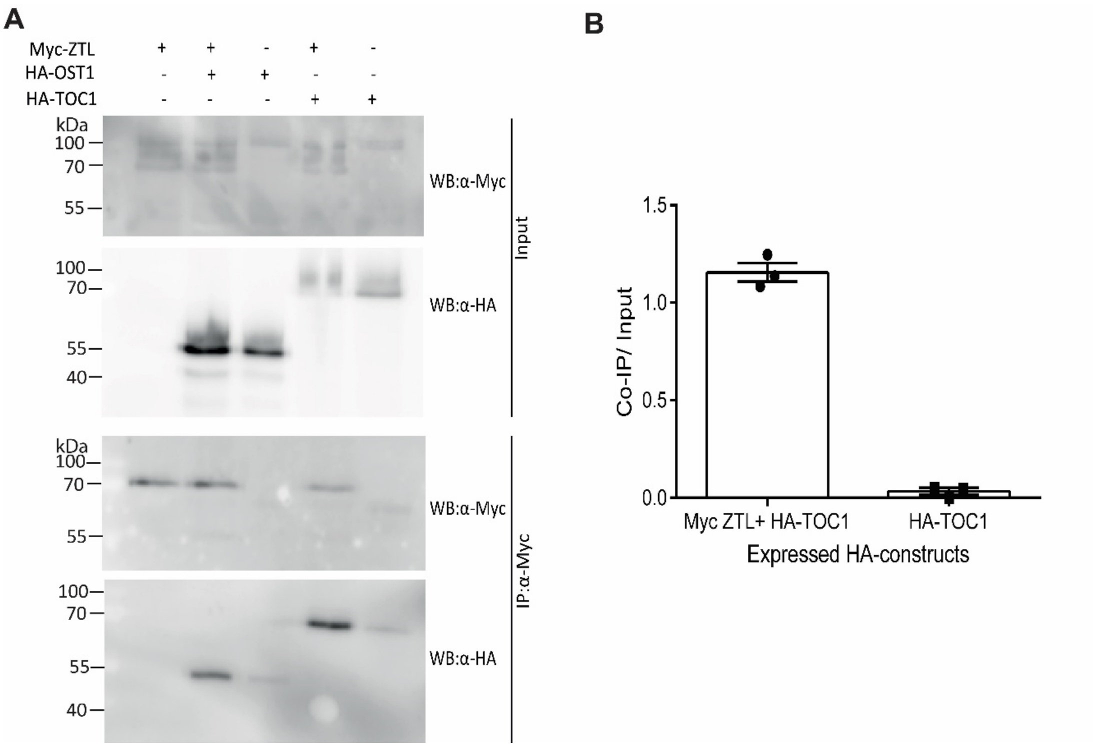

Supplementary Figure 6. Interaction between ZTL and TOC1 *in vivo*. (**A**) Myc-ZTL and HA-TOC1 were co-expressed in Arabidopsis protoplasts and the interaction between the proteins determined by co-immunoprecipitation. (**B**) The ZTL-TOC1 interaction signal is strong in protoplasts. Data from three independent experiments where the ratio of Co-IP signal to sample signal from the input reaction (40% of sample used in Co-IP) run in separate Western blots. Means of three experiments (n=3) ± SEM are shown, with data points from each experiment plotted for Myc-ZTL + HA-TOC1 (left bar, circles) and HA-ZTL (right bar, squares). Student's t-tests showed significant differences at $\alpha < 0.05$, $n = 3$, between Myc-ZTL + HA-TOC1 and HA-TOC1 ($P < 0.0001$).

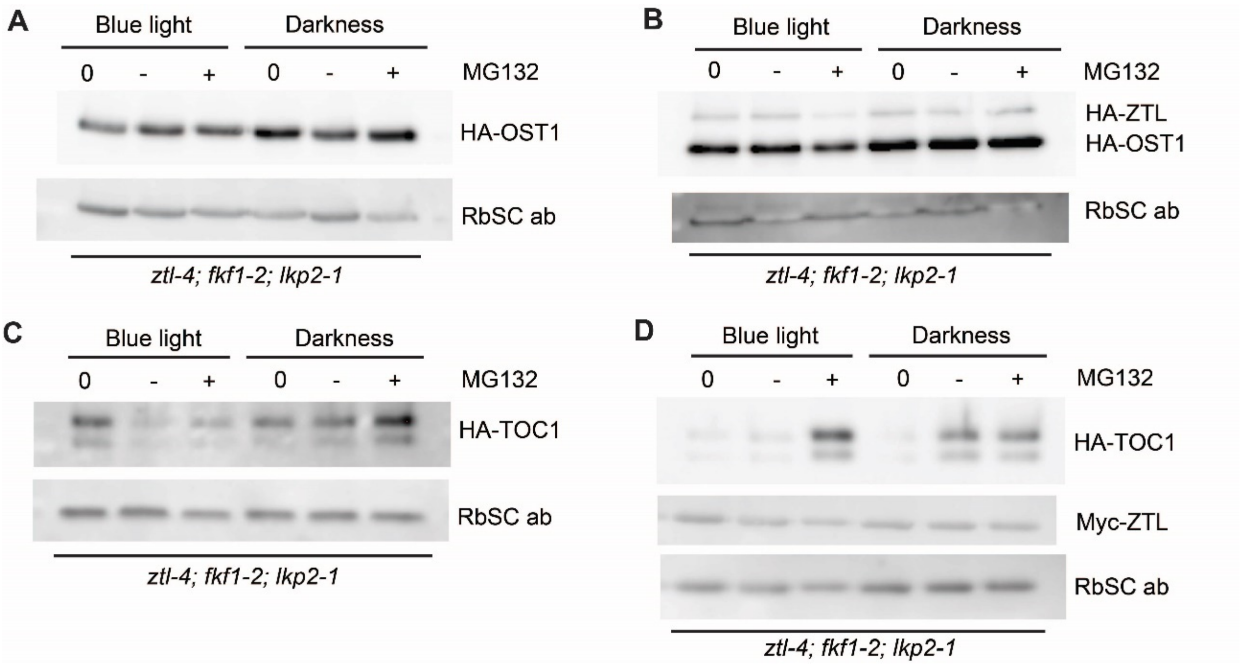

Supplementary Figure 7. ZTL enhances TOC1 degradation. Arabidopsis mesophyll protoplasts from the *ztl-4; fkf1-2; lkp2-1* triple mutant were transfected with (**A**) HA-OST1; (**B**) HA-ZTL and HA-OST1; (**C**) HA-TOC1; (**D**) Myc-ZTL and HA-TOC1. Protoplasts were transfected with expression vectors, incubated for 4 h and treated with MG132 or DMSO (time 0). Protoplasts were then incubated for a further 4 h before proteins were immunoprecipitated. The entire experiment was performed twice, once under blue light, once in darkness. RbCS (Rubisco small subunit antibody; lower panels **A–D**) was used as a loading control for all immunoblots.

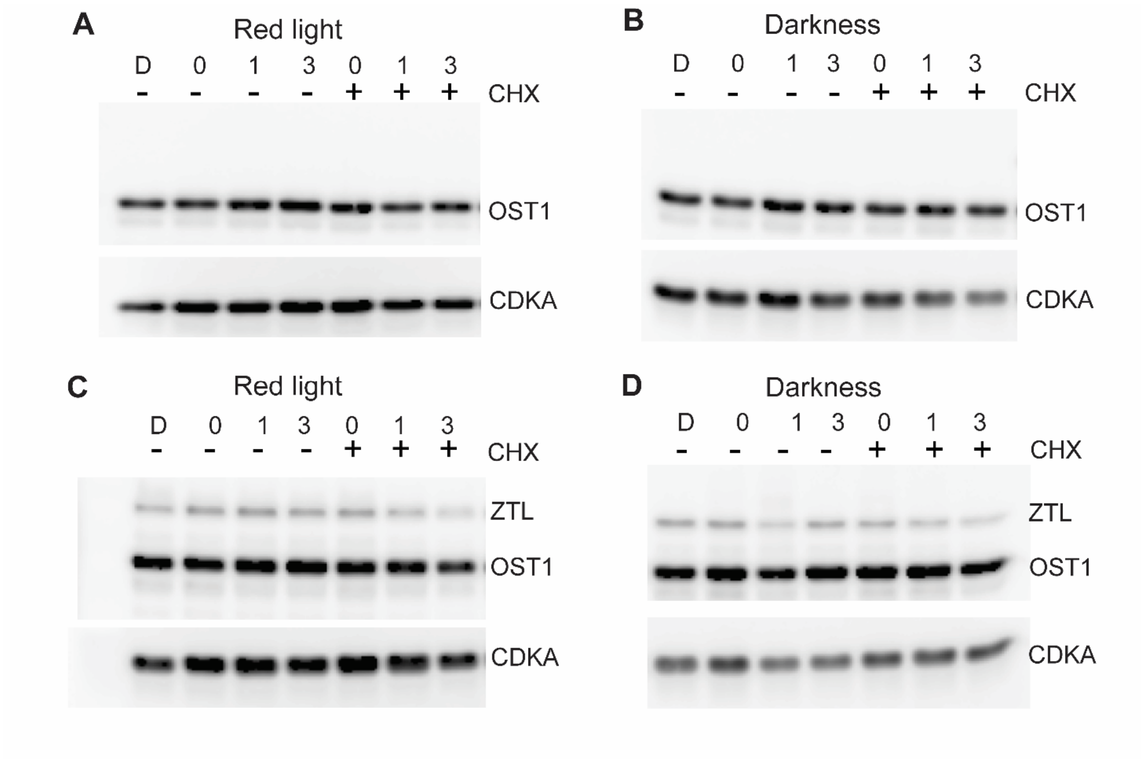

Supplementary Figure 8. ZTL does not target OST1 for degradation. Arabidopsis cell suspension protoplasts were transfected with (**A, B**) HA-OST1 or (**C, D**) HA-OST1 and HA-ZTL. Protoplasts were incubated in the dark for 18 hours post-transfection before the addition of 100 µM CHX to block protein expression (+) or vehicle control (-). Samples were then (**A, C**) exposed to 10 µmol m^{-2} s^{-1} red light or (**B, D**) maintained in darkness. ZTL and OST1 expression levels were measured 0, 1 and 3 hours after CHX treatment. In (**A-D**) a control sample (marked 'D' on lane labels) remained in the dark throughout. CDKA;1, PSTAIRE antibody was used as a loading control (**A-D** lower panels).

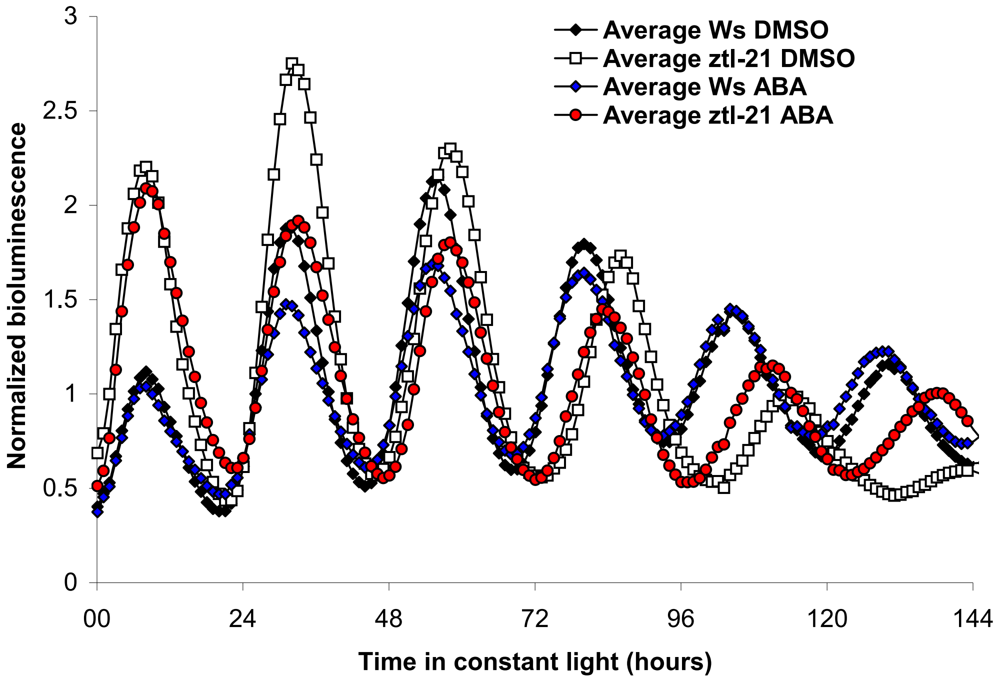

Supplementary Figure 9. Representative CAB2:LUCIFERASE (LUC) data from Ws-2 and *ztl-21* Arabidopsis assayed under blue light ± ABA. Seedlings were entrained in light:dark (LD) 12:12 cycles for 7 days, then transferred to 0.5 MS plates without sucrose ± 20 µM ABA in 0.01 % DMSO. After an additional LD cycle, plants were exposed to constant blue light (15 µmol m2 s-1) for recording of LUC activity. Period analyses of these data are shown in Supplementary Table 1.

Supplementary Table 1: Effect of ABA treatment on period length under blue light (data in Figure 1).

Genotype	Treatment	Period (h)	SEM	Rhythmic n
Ws-2	DMSO	24.5	0.3	20
Ws-2	ABA	24.4	0.1	20
ztl-21	DMSO	27.0	0.1	20
ztl-21	ABA	25.9	0.2	20

Supplementary Table 2. Effect of ABA treatment on period of CAB2:LUCIFERASE (LUC) activity in artificial 'white' light. Seedlings were entrained in light:dark (LD) 12:12 for 7 days, then transferred to 0.5× MS plates without sucrose ± 20 µM ABA in 0.01% DMSO. After an additional LD cycle, plants were moved to constant light (red + blue, each 10 µmol m2 s-1) for recording of LUC activity.

Genotype	Treatment	Period (h)	SEM	Rhythmic n
Ws-2	DMSO	24.9	0.2	20
Ws-2	ABA	24.1	0.2	20
ztl-21	DMSO	26.2	0.1	20
ztl-21	ABA	25.4	0.3	20

Supplementary Material

Lists of primer sequences used in experiments

1. Primer sequences used in *Populus tremula* × *P. tremuloides* (*Ptt*) RNAi gene constructs

Primers include Gateway sequences (underlined)

PttZTL-F: 5′-GGG GAC AAG TTT GTA CAA AAA AGC AGG CCA TTG AAT TCC AAG GTG AGT-3′

PttZTL-R, 5′-GGG GAC CAC TTT GTA CAA GAA AGC TGG GTA GCA TTC TGA CAG ACC ATT C-3′

2. Primers used for PCR colony of pGreenII0229-ZTL Arabidopsis construct

Primers include restriction sites (underlined)

ZTL-BamHI-F: 5´-GCA GGA TCC ATG GAG TGG GAC AGT GGT TC-3´

ZTL-SalI-R: 5´-GCA GTC GAC CTA ATG AGG AAG AAA GAA GAA GAA G-3´

3. Primer sequences for production of Arabidopsis protein expression vectors

Restriction enzyme recognition sequences (underlined) were added to primers to enable cloning of amplified fragments into the matching sites in the epitope-tagged pRT104 vector.

OST1-EcoRI-F: 5′-GCA GAA TTC ATG GAT CGA CCA GCA GTG AGT-3′

OST1-SalI-R: 5′-GCA GTC GAC TCA CAT TGC GTA CAC AAT CTC-3′

PRR5-BamHI-F: 5′-GCA GGA TCC ATG ACT AGT AGC GAG GAA GTA G-3′

PRR5-KpnI-R: 5′- GCA GGT ACC CTA TGG AGC TTG TGT GGA TTG-3′

SKIP32-BamHI-F: 5′-ATA GGA TCC GAT GCT CTC ACT ATA CCA-3´

SKIP32-XhoI-R: 5′ CCC TCG AGT TAG CCA GTG ACA TAG TAA TC-3′

4. Primers used in quantitative reverse transcription PCR (RT-qPCR) using Arabidopsis

ABF3-F: 5′-GTG CAG TTC TGG AGA AAG TG-3′

ABF3-R: 5′-CAA TTT CTG CTT CCA GTT CCA-3′

ABF4-F: 5′-TAA TCG AGG ACG AAG AAG CA-3′

ABF4-R: 5′-TCC AGT TCC AAT GTA TAA GCC T-3′

ABI2-F: 5′-CAT TGG CGA TAG ATA CCT TAA ACC-3′

ABI2-R: 5′-CAT TAC ATC CCA AAG ACC ATC AC-3′

ABI5-F: 5′-GAG AGG AAG AGG AAG CAA CAG-3′

ABI5-R: 5′-ATC AAT GTC CGC AAT CTC CC-3′

EF1a-F: 5′-TGA GCA CGC TCT TCT TGC TTT CA-3′

EF1a-R: 5′-GGT GGT GGC ATC CAT CTT GTT ACA-3′

HAB1-F: 5′-GTT CTC GCC ATG TCT AGG TC-3′

HAB1-R: 5′-CAT CAA TAT CCG TCT CCT TGC T-3′

OST1-F: 5′-CCA CTC AGT TTG ATG AAT CGG-3′

OST1-R: 5′-TAT GTC CAA GCT TCC TGT GAG-3′

PYL5-F: 5′-CCG ATG GTC CGA TCA AGA G-3′

PYL5-R: 5′-CGA GGA GCA ACA CTG GTC-3′

RAB18-F: 5′-GGA GGA TGA TGG ACA AGG AG-3′

RAB18-R: 5′-TTG ACC AGA CTG ATC ATG ATG AC-3′

RD29A-F: 5′-CTT GAT GGT CAA CGG AAG GT-3′

RD29A-R: 5′-CAA TCT CCG GTA CTC CTC CA-3′

5. Primers used in RT-qPCR using *P̲opulus t̲remula* × *P. t̲remuloides* (*Ptt*)

PttZTL1,2-F: 5’-5’CTG GAG CGG AAC CGA AGG3’-3’

PttZTL1,2-R: 5’-5’GTCAATGGAGAAGCCTATCTGG3’-3’

EF1a-F and -R (as above)

18S rRNA-F: 5’- TCA ACT TTC GAT GGT AGG-3’

18S rRNA-R: 3’- CCG TGT CAG GAT TGG GTA ATT T-3’
